# Mass Mortality Caused by Highly Pathogenic Influenza A(H5N1) Virus in Sandwich Terns, the Netherlands, 2022

**DOI:** 10.3201/eid2812.221292

**Published:** 2022-12

**Authors:** Jolianne M. Rijks, Mardik F. Leopold, Susanne Kühn, Ronald in ‘t Veld, Fred Schenk, Allix Brenninkmeijer, Sander J. Lilipaly, Mónika Z. Ballmann, Leon Kelder, Job W. de Jong, Wouter Courtens, Roy Slaterus, Erik Kleyheeg, Sandra Vreman, Marja J.L. Kik, Andrea Gröne, Ron A.M. Fouchier, Marc Engelsma, Mart C.M. de Jong, Thijs Kuiken, Nancy Beerens

**Affiliations:** Dutch Wildlife Health Centre, Utrecht University, Utrecht, the Netherlands (J.M. Rijks, M.J.L. Kik, A. Gröne);; Wageningen Marine Research, Den Helder, the Netherlands (M.F. Leopold, S. Kühn);; Staatsbosbeheer Zuid-Hollandse Delta, Numansdorp, the Netherlands (R. in ’t Veld);; Stichting Het Zeeuwse Landschap, Wilhelminadorp, the Netherlands (F. Schenk);; Province of Groningen, Groningen, the Netherlands (A. Brenninkmeijer);; Deltamilieu Projecten, Vlissingen, the Netherlands (S.J. Lilipaly, M.Z. Ballmann);; Staatsbosbeheer Beheereenheid de Kop, Schoorl, the Netherlands (L. Kelder);; Bureau Waardenburg, Culemborg, the Netherlands (J.W. de Jong);; Research Institute for Nature and Forest, Brussels, Belgium (W. Courtens);; Sovon Dutch Centre for Field Ornithology, Nijmegen, the Netherlands (E. Kleyheeg, R. Slaterus);; Wageningen Bioveterinary Research, Lelystad, the Netherlands (S. Vreman, M. Engelsma,, N. Beerens);; Department of Viroscience, Erasmus MC, Rotterdam, the Netherlands (R.A.M. Fouchier, T. Kuiken);; Wageningen University and Research, Quantitative Veterinary Epidemiology group, Wageningen, the Netherlands (M.C.M. de Jong)

**Keywords:** influenza A virus, H5N1 subtype, wild animals, influenza in birds, Charadriiformes, seasons, breeding, disease outbreaks, primary disease prevention, viruses, the Netherlands, *Thalasseus sandvicensis*, Sandwich terns, influenza, respiratory infections, zoonoses

## Abstract

We collected data on mass mortality in Sandwich terns (*Thalasseus sandvicensis*) during the 2022 breeding season in the Netherlands. Mortality was associated with at least 2 variants of highly pathogenic avian influenza A(H5N1) virus clade 2.3.4.4b. We report on carcass removal efforts relative to survival in colonies. Mitigation strategies urgently require structured research.

The 2021–2022 epidemic of highly pathogenic avian influenza (HPAI) A(H5N1) virus clade 2.3.4.4b has been unprecedented in terms of numbers of dead wild birds, species affected, spatial extent, and incidence in spring 2022 (*1*). Across Europe, multiple colony-breeding seabirds experienced HPAI H5N1–associated mass mortalities during the breeding period, including the Sandwich tern (*Thalasseus sandvicensis*) (*1*,*2*). The Netherlands constitutes a major though vulnerable stronghold of the Sandwich tern in Europe; 15,000–20,000 breeding pairs have been documented across ≈10 colonies (https://stats.sovon.nl/stats/soort/6110). We sought to establish the scale of mortality occurring in Sandwich terns breeding in the Netherlands in 2022, characterize the associated HPAI H5N1 viruses and pathology, report on the carcass removal effort relative to survival, and investigate intracolony transmission dynamics.

## The Study

We determined breeding colony locations and initial sizes in May 2022 through drone or ground counts (*3*). To establish breeding success and minimum estimates of mortality, we compiled data from late May through early July 2022 on numbers of live adults, chicks, fledglings, and late clutches in colonies, as well as on numbers of carcasses found in and around colonies, carcasses removed for destruction, and abandoned nests (Appendix 1 Table 1). Sandwich terns lay 1–2 eggs and incubate for 21–29 days. Chicks fledge 25–30 days after hatching; annual fledging success is ≈0.5 per breeding pair (*4*–*6*). We used wild bird mortality databases to establish minimum estimates of adult mortality outside the colonies. Finally, we used data from the migration tracking website Trektellen (https://www.trektellen.nl) to compare the hourly averages of Sandwich tern passing rates at coastal observation points per week in 2022 to 2016–2021.

We observed clinical signs and tested 44 carcasses for avian influenza virus by using a quantitative PCR to detect the influenza A virus matrix gene; we then followed up with subtype-specific PCRs on cloacal and tracheal swab specimens (*7*). We performed necropsy with histopathology and immunohistochemistry on 6 of the carcasses to establish cause of death. To study the relationship between viruses detected in Sandwich terns and other bird species, we determined full-genome sequences directly on swab RNA from 20 birds and submitted them to the GISAID database (https://www.gisaid.org), then compared them to a sample of 57 other birds (Appendix 1 Table 2). We aligned sequences by using MAFFT version 7.475 (*8*), reconstructed phylogeny by using maximum-likelihood analysis with IQ-TREE software version 2.0.3 (*9*), and visualized the maximum-likelihood tree by using the R package ggtree (*10*).

To investigate HPAI H5N1 transmission dynamics within a breeding colony, we developed a susceptible-infectious-recovered model that included infection-fatality rate (IFR) and examined outcome as a function of IFR. IFR is the probability that a bird dies after infection, although in this study IFR also includes birds leaving the colony. The model assumed a naive starting population, an infectious period of 5 days, and frequency-dependent transmission and considered only survivors of infection as recovered and total population size as not constant (Appendix 2).

**Figure 1:**
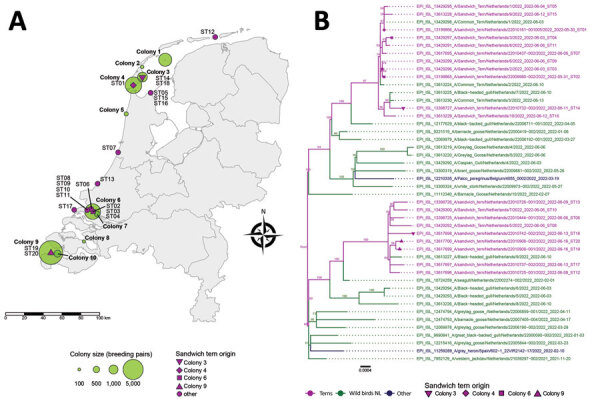
Location of Sandwich terns affected by locally acquired highly pathogenic avian influenza A(H5N1) clade 2.3.4.4b viruses and phylogeny of viral segments, the Netherlands. A) Location and size (number of breeding pairs) of the Sandwich tern breeding colonies and the origin (finding location) of the Sandwich terns from which virus sequences ST01–ST20 shown in the phylogenetic tree in panel B were obtained. B) Maximum-likelihood tree (1,000 bootstraps) of the concatenated viral segments showing the H5N1 viruses detected in Sandwich terns together with viruses from other wild birds. Bootstrap values >50 are indicated at the branches. Identification numbers and symbols of the Sandwich terns correspond to those in the map, and the date that the bird was found dead is indicated. The GISAID sequences used in the phylogenetic analysis are listed in Appendix 1 Table 2. ST, Sandwich tern.

Mass mortality was seen in 9 of the 10 Sandwich tern breeding colonies in 2022. In those colonies, out of a total of 18,151 breeding pairs, 8,001 adult Sandwich terns were found dead, and only a few chicks fledged (Video 1; Video 2). Only 1 small inland colony of 137 breeding pairs experienced no mass mortality and had a fledgling success rate (0.47 young/pair) consistent with previous years (0.50 young/pair) (Table; Figure 1, panel A). Outside of colonies, another 1,600 adult Sandwich terns were reported dead between late May and end of June. The scale of mortality is reflected in the passage rate of Sandwich terns along the coast in May–June 2022 (Appendix 2 Figure 4).

**Video 1:** Video clip taken while removing dead birds from Colony 6 (Slijkplaat) on June 9, 2022 (9 days after first dead birds were observed in this colony), the Netherlands. Amid the many dead adult Sandwich terns, 1 live bird with spread-out wings was breathing heavily and showing opisthotonos, flipping over backward when trying to move away from camera. This bird died shortly after the video was taken and was removed from the colony, together with 120 other dead adult birds on that day (Appendix 1 Table 1). Video credit: Ronald in ‘t Veld.

**Video 2:** Video clip taken in colony 3 (Wagejot) on June 9, 2022 (5 days after the first dead birds were seen in the colony), the Netherlands. Many dead chicks littered the colony, 1 in the shelter of the wing of a dead adult Sandwich tern (presumably 1 of its parents). That day, hundreds of dead chicks were reported (Appendix 1 Table 1). Video credit: Eckard Boot, Natuurmonumenten.

**Table:** Mortality, fledgling success, carcass removal, and estimated fraction of Sandwich tern breeding population that died or disappeared in 10 breeding colonies, the Netherlands, 2022

Breeding colony no.	Date mortality first observed	Fledgling success (no. fledglings produced/no. initial breeding pairs)	Dead adults found	Dead chicks found	Abandoned eggs	Carcass removal	Estimated percentage of breeding population that died or disappeared from colony*
Colony 1	2022 May 30	1% (20/2,100)	342	425	Yes, all 700 late nests	Yes, with a 12-d delay, on 10 of next 18 d, adults and chicks	99%
Colony 2	2022 Jun 21	0% (0/150)	107	0	Yes, all 150 late nests	Once, with a 3-d delay, adults only	100%
Colony 3†	2022 Jun 4	0% (0/1,176)	170	Hundreds	Yes, many	Yes, with a 6-d delay, on 6 of next 18 d, adults only	100%
Colony 4†	2022 May 26	0.1% (5/3,374)	3,316	Thousands	Unknown	Twice, once 19 d after start, then 16 d later, adults only	99.8%
Colony 5	2022 May 29	0% (0/220)	400	0	Unknown	Once, 17 d after deaths started, adults only	100%
Colony 6	2022 May 31	Low (Few/3,016)‡	941	Thousands	Yes, all 245 late nests	Yes, without delay, on 15 of next 36 d, adults only	>92%
Colony 7	2022 May 31	11.1% (45/404)	115	Unknown	Yes, many of 40 late nests	Yes, without delay, on 14 of next 36 d, adults only	78%
Colony 8	2022 Jun 14	47.4% (65/137)	2	0	No (also no late nests)	No	5% or not applicable§
Colony 9	2022 Jun 6	9.5% (665/6,974)	2,368	3,122	No, there is activity above 400 late nests	Yes, with an 8-d delay, on 14 of next 27 d, adults and chicks	81%
Colony 10¶	2022 Jun 14	0% (0/600)	240	12	Unknown, 150 + 450 late nests	Yes, with a 7-d delay, on 3 of next 15 d, adults and chicks	100%
Overall		Low (Few/18,151 pairs)	8,001 (22% of breeding birds)	Thousands			

*Calculated by subtracting the ratio of fledgling success in 2022 over average fledgling success in previous years (0.5 fledgling per pair) from 100%. †After colony 3 and colony 4 were decimated, a very late colony of 600 breeding pairs was established in July on Texel midway between these 2 colonies (53.022°N, 4.819°E), and this very late colony yielded 300 fledglings in September 2022. ‡Exact number not recorded. §Because no carcass was examined, it is unclear if the 2 dead birds observed in this colony died from other causes or the cause of death was HPAI H5N1 but further infections in the colony were aborted. ¶In this colony, some very late birds did end up producing some fledglings (around 155) in August (Appendix 1 Table 1).

Diseased birds were debilitated, unable to fly, and mostly lethargic, sometimes with wings spread out. At later stages, some displayed opisthotonos and occasionally flipped over backwards (Video 1, Video 3; Appendix 2 Figures 5–10). We confirmed HPAI H5N1 virus infection in 23 of 24 dead Sandwich terns from colonies (the exception was a chick); infection was also confirmed in 20 of 20 birds outside of colonies (Appendix 2 Table 1). In the 4 necropsied PCR-confirmed adult birds, viral antigen expression was detected by immunohistochemistry in the pancreas (n = 3), duodenum (n = 4), or lung and nasal tissue (n = 1), colocalized with necrosis and inflammation (Appendix 2 Figure 18). Necropsy findings and negative immunohistochemistry results in the 2 chicks we examined demonstrated that chick mortality was at least partly caused by starvation, likely after feeding was interrupted because of adult mortality (Appendix 2). This explanation was supported by field observations (Appendix 2 Figure 19).

**Video 3:** Webcam sequence of clips from within colony 9 (Waterdunen), June 15, 2022 (10 days after first dead birds were observed in this large colony), the Netherlands. 07:51: Many adults respond to unseen disturbance by flying off, but 1 sick bird remains, unable to fly, tries to cover its chick with its wing but starts faltering. 07:56: Other adults have returned, including presumed partner of sick bird. Chick turns to other parent and stumbles over sick bird. Partner pecks at sick bird, driving it away. Sick bird staggers away and takes cover at nearby plant. 08:07. Sick bird no longer moves. Chick and partner pass by, without further interaction, leaving bird (presumably dead) as is. In total, >900 dead Sandwich terns were removed from this colony on June 14 and 15, 2022 (Appendix 1 Table 1). Webcam credit: Bureau Waardenburg and Stichting Het Zeeuwse Landschap.

Phylogenetic analysis demonstrated that the 20 fully sequenced viruses belonged to H5 clade 2.3.4.4b, and clustered with viruses detected in other wild bird species in the Netherlands, including in geese and gulls collected during January–April 2022 (Figure 1, panel B; Appendix 2). The Sandwich tern viruses clustered in 2 groups. Both variants were found in the northern and southern parts of the Netherlands and were even found within a single colony. These results suggest that at least 2 independent virus introductions into Sandwich terns occurred in the Netherlands, followed by transmission of both virus variants within and between breeding colonies.

Carcass removal effort was diverse (Table; Appendix 1 Table 1). In colonies with some survival, effort was overall more regular, frequent, and immediate or included chicks also, although survival might also have been affected by other undetermined factors.

**Figure 2:**
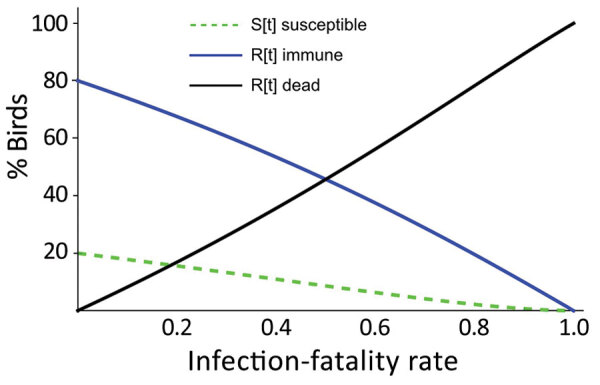
Model result for the introduction of highly pathogenic avian influenza (HPAI) A(H5N1) clade 2.3.4.4b viruses into a local Sandwich tern breeding colony population, the Netherlands. Graph demonstrates the distribution at the end of the local HPAI H5N1 outbreak, for dead (or departed) birds, escaping susceptible birds, and immune birds as a function of the infection-fatality rate (IFR), for a Sandwich tern population that was naive for HPAI H5N1 at the start of the local outbreak, for R_0_ = 2. The model output indicates that the fraction of birds infected with HPAI H5N1, and hence also the fraction that dies, will increase with infection-fatality rate and that at a rate above 90%, virtually no more susceptible birds remain, only immune or dead (or departed) birds.

In most colonies, a high proportion of the birds died or left (Table), indicating high IFR. The model shows that, even with a relatively low value of R_0_ (R_0_ = 2), at the higher end of the IFR range evidenced here, few of the birds remaining in the colony will have escaped infection at the end of the outbreak. Birds in the colony will have died or recovered and acquired immunity (Figure 2).

## Conclusions

Our results substantiate that after Sandwich terns arrived in the Netherlands for breeding, HPAI H5N1 virus was introduced into their population at least twice. The virus then spread widely within and between breeding colonies, causing outbreaks that resulted in high adult and chick mortality in nearly all colonies. Infected birds probably died of systemic HPAI-associated disease, including acute pancreatic necrosis and duodenitis (*11*,*12*). Like other seabirds, Sandwich terns have low annual reproductive output but relatively long life-expectancy (*2*,*4*,*6*,*13*); therefore the effect of high adult mortality on population size could be seen for a long time. The Sandwich tern exemplifies how severely the continued circulation of HPAI H5N1 viruses in spring 2022 affected populations of colony-breeding birds without flock immunity in Europe (*1*).

Our study also demonstrates how outbreaks in breeding birds boosted virus propagation into the summer of 2022. The future involvement of Sandwich terns in HPAI endemicity can be evaluated once future population size and flock immunity have been analyzed from count data and serosurveillance. On the basis of our model, colony survivors would be mostly immune to HPAI.

Confirming HPAI as a major mortality factor in breeding colonies of Sandwich terns and other seabird species (*2*,*14*,*15*) underlines the paradigm shift to HPAI as a mortality factor of concern to wild species, in addition to poultry and humans. It stresses the importance of close international cooperation and data exchange to better understand and mitigate the global effect of HPAI on nature. More structured research on appropriate strategies to reduce massive propagation is urgently required. Carcass removal takes away a source of infection but might simultaneously enhance spread of infection and thus requires controlled study.

Appendix 1 Additional data from study of mass mortality caused by highly pathogenic influenza A(H5N1) virus in Sandwich terns, the Netherlands, 2022

Appendix 2 Additional information about mass mortality caused by highly pathogenic influenza A(H5N1) virus in Sandwich terns, the Netherlands, 2022
