# Supplementary figures and images for: Mass Mortality Caused by Highly Pathogenic Influenza A(H5N1) Virus in Sandwich Terns, the Netherlands, 2022
Source: Emerg Infect Dis. 2022 Dec;28(12):2538–42. doi: 10.3201/eid2812.221292 (PMC9707584; doi:10.3201/eid2812.221292)

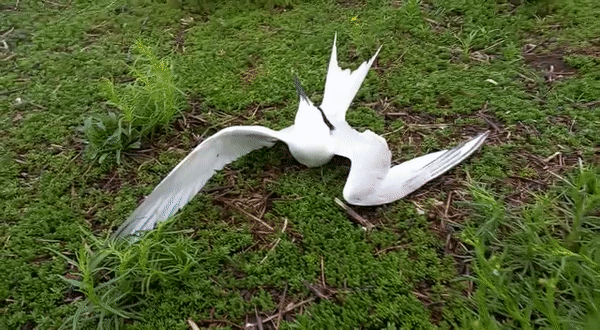

Supplement: Supplementary file 1 [file 22-1292-V1.gif]

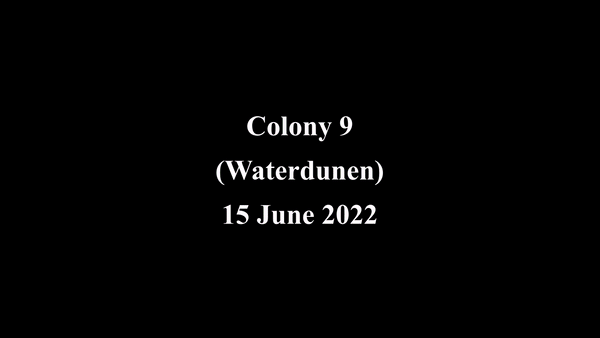

Supplement: Supplementary file 3 [file 22-1292-V3.gif]
