# Supplementary material for: Mass Mortality Caused by Highly Pathogenic Influenza A(H5N1) Virus in Sandwich Terns, the Netherlands, 2022
Source: Emerg Infect Dis. 2022 Dec;28(12):2538–42. doi: 10.3201/eid2812.221292 (PMC9707584; doi:10.3201/eid2812.221292)
Supplement: Appendix 2 — Additional information about mass mortality caused by highly pathogenic influenza A(H5N1) virus in Sandwich terns, the Netherlands, 2022 [file 22-1292-Techapp-s2.pdf]

Article DOI: https://doi.org/10.3201/eid2812.221292

Mass Mortality Caused by Highly Pathogenic Influenza A(H5N1) Virus in Sandwich Terns, the Netherlands, 2022

Appendix 2

Model

This section presents the model of the effect of infection-fatality rate (IFR) on HPAI H5N1 transmission dynamics in a breeding colony with an initially naive Sandwich tern (*Thalasseus sandvicensis*) population.

Specifics

This analysis focuses on the breeding colonies as clearly defined local populations. The birds in these breeding colonies are special in that they have a strong tendency to remain there to maximize their fitness. Breeding birds will disappear from a local population only because they die or because they leave after breeding failure.

As explained in the main article, the observed Sandwich tern mortality is considered to occur after introduction of the virus into a local breeding colony population consisting completely of naive individuals. The outbreaks are remarkable because a very high IFR appears to have occurred during these outbreaks, but the massive drops in numbers may partly be due to birds abandoning their nest after their partner and chicks died. The IFR is the probability that a bird dies when it has become infected but, in this model, it may also account for birds leaving the breeding colony population alive.

Model

The following Susceptible-Infectious-Recovered (SIR) model, which is a simplified but possibly relevant representation of the reality, looks at the outbreak in a local population (a breeding colony population of Sandwich terns) completely naive for the infecting H5N1 influenza strain, and considers the effect of IFR on the dynamics.

$$\frac{d\,S(t)}{dt} = -\beta \frac{S(t)I(t)}{S(t)+I(t)+R(t)}$$

$$\frac{d\,I(t)}{dt} = \beta \frac{S(t)I(t)}{S(t)+I(t)+R(t)} - \alpha I(t)$$

$$\frac{d\,R(t)}{dt} = (1 - IFR)\,\alpha I(t)$$

This formulation looks very much like the standard SIR model with the transmission term being divided by the population size assuming therefore frequency-dependent transmission (*1*). Two parts are special for this SIR model: 1) the total population size is not constant hence it is written as the sum of the numbers of susceptible birds, infectious birds and recovered birds, and 2) the recovered birds here are the only (surviving) birds that become immune as can be seen from the last equation.

Transmission (R_0) of this Highly Pathogenic Avian Influenza (HPAI) in Sandwich terns in a breeding colony does not need to be that high to obtain the observed pattern. We used a conservative value of $R_0 = 2.0$ here. Any other (higher) value would not have changed the result reported in the main text (see below). Observations on the final size as function of IFR would allow us to estimate the R_0. The final size is the final outcome of the introduction of the virus to the local breeding colony population: i.e., the number of birds that died, that recovered and that remained susceptible (escaped infection). For example, in most of the 9 affected breeding colonies mortality (or in some cases departure) seemed to be near 100%, and in a few others around 80% (Table 1). The first value implies IFR = 1.0 (100%) and could be any $R_0>1$. The second value implies for $R_0 = 2.0$ that IFR = 0.8156 (82%) and apart from the 80% dead or otherwise disappeared birds, most of the surviving birds will be immune (19%) and the remaining 1% of birds will have escaped infection. For higher values of R_0 all surviving birds will also be infected and thus will be recovered and immune.

If IFR = 0 than for the parameters chosen here, i.e., $R_0 = 2.0$ and the duration of the infectious period is 5 days (thus, $\beta = 0.4\ day^{-1}$ and $\alpha = 0.2\ day^{-1}$) on average 20% will escape infection in a major outbreak (Appendix 2 Figure 1) and the remaining 80% will become immune.

If IFR = 1, i.e.,100% mortality if infected, then with the same parameters more individuals will become infected (here all individuals as IFR = 1) and hence also more individuals will die. Given IFR = 1 all individuals will die (Appendix 2 Figure 2).

In the main article the Final Size for the different type of birds as a function of IFR is given for $R_0 = 2.0$. Choice of a higher value for R_0 will only change the fraction of the surviving birds that is immune rather than escaped infection. For example, for $R_0 = 6.0$ the figure in the main text would look like Appendix 2 Figure 3.

Discussion of Assumptions

The model used has the following assumptions, each followed by the argumentation why this assumption was used:

1. Not stochastic: the calculation just shows the average. In the situation considered here IFR is almost 1.0, the average of close to 100% infected and also close to 100% dying there is little variation even in the stochastic model. As in 9 of 10 breeding colonies that seems what has happened the calculation seems in agreement with the data. Because after dying of the original breeding birds, other conspecific birds will attempt to establish themselves and thus one cannot distinguish whether mortality is 100% or close to 100%.

2. Survival of the virus in the environment is not taken into account: in the model the recovery rate parameter $\alpha = 0.2$ day^{-1}: individuals are expected to be infectious for on average 5 days (1/0.2) with an exponential distribution. In reality birds may die earlier but infectious material, i.e., virus, will be present in the environment. In a similar model for breeding black-headed-gulls, a similar value, i.e., 0.26 day^{-1}, was used for the exponential decay rate parameter (Verhagen, Fouchier, and de Jong in prep.).

3. Crucial assumption is that the transmission rate is frequency dependent which means that the contact rate is constant for different population sizes. Here the population size decreases and thus for the remaining birds the chances of encountering the infectious birds and infectious material (shed before death or dead birds) increase. This is the case because with high IFR all birds in a population are either susceptible or infectious and not immune individuals. This seems a reasonable assumption, as all the area where the breeding birds have died will have a lot of infectious material and breeding birds will continue taking care of the nest and the chicks in this contaminated environment.

4. The population was initially immunologically naïve for HPAI H5N1. This assumption is based on the lack of reports on HPAI die-offs in Sandwich terns in previous years (*2*).

Passing Rate

The scale of Sandwich tern mortality is reflected in the decreased hourly average passing rate at coastal observation points in the Netherlands, June through August 2022, as compared to 2016–2021 (Appendix 2 Figure 4). It should be noted that observation effort (hours) is not equally distributed along the coast and that locations near hard-hit colonies may be overrepresented in this graph. Source data: www.trektellen.nl (*3*).

Clinical Signs, Postmortem Postures, and Outbreak Features

Diseased birds were debilitated, unable to fly, mostly lethargic, sometimes with wings spread out. At later stages some displayed opisthotonos, while occasionally flipping over backward, sometimes dying in that position (Appendix 2 Figures 4-14). Necrophilia may have enhanced transmission among adults (Appendix 2 Figure 15). Carcass removal around and away from colonies required organization and came with logistical challenges (Appendix 2 Figures 16, 17).

PCR Test Results

Overview of the dead Sandwich terns tested for avian influenza by PCR, with outcome and virus sequence number if sequencing performed (Appendix 2 Table 1). Those also submitted for necropsy with histopathology and immunohistochemistry are indicated.

Pathology and Immunohistochemistry

Here we present the results of the necropsies with histopathology and immunohistochemistry on 4 adult and 2 chick Sandwich terns.

Adults

Four dead adult Sandwich terns, 1 from Engelsmanplaat and 3 from Breeding Colony 3 (Wagejot), were evaluated for pathologic changes and tissue related virus protein expression. All

four birds had tested positive for HPAI H5N1 by PCR-tests, and 2 birds were also sequenced (Appendix 2 Table 2). Gross pathology showed the carcasses were moderately autolytic. All birds were in a good body condition; however, the stomach was empty, and the intestinal tract was poorly filled. The livers were enlarged, extending 2–3 cm below the carina. Spleens were not enlarged (0.5cm x 0.5cm). (Appendix 2 Table 3). Histopathology and immunohistochemistry showed the most prominent finding in all birds was the severe acute pancreas necrosis (Appendix 2 Figure 18, panel A) with associated viral antigen expression in cells (Appendix 2 Figure 18, panel B), which was present in 3 of the 4 birds. All birds showed a mild to moderate non-suppurative duodenitis (Appendix 2 Figure 18, panel C), which was also associated with viral antigen expression (Appendix 2 Figure 18, panel D). The lungs showed mainly a-specific changes, such as edema and congestion; only in one bird was there mild virus expression in the lung and nasal/sinus tissue. There was no evidence for infiltrates of inflammatory cells or virus expression in the cerebrum and cerebellum, and also the other organs showed no significant histopathologic findings (nor virus expression), which could be associated with another (infectious) cause of death (Appendix 2 Tables 3, 4).

By this we conclude that the HPAI infection in the examined adult Sandwich terns was associated with severe pancreas necrosis and duodenitis and less prominent changes in the respiratory tract and other investigated organs. Acute pancreas necrosis has also been reported in other bird species infected with this virus clade (*4*).

Chicks

Two dead Sandwich tern chicks from Breeding Colony 6 (Slijkplaat) were evaluated for pathologic changes and tissue related viral antigen expression. The larger chick had tested positive for HPAI H5N1 by PCR-tests, while the smaller had tested negative (Appendix 2 Table 2). Gross pathology showed the stomachs were empty (Appendix 2 Table 3). Histopathology and immunohistochemistry showed the lesions in the chicks were not associated with viral antigen expression. At least in the smaller chick, death by dehydration/starvation could be probable (Appendix 2 Tables 3, 4).

Field Observations on Timing of Adult Mortality Versus Chick Mortality

Field observations showed live chicks among dead adults (Appendix 2 Figure 12). Counts of dead adults and dead chicks over time support pathology that indicates that chick mortality was at least partly due to starvation following interrupted feeding because of adult HPAI-H5N1-associated mortality (Appendix 2 Figure 19).

Phylogentic Trees

The maximum-likelihood (ML) tree of the 8 virus segments PB2, PB1, PA, HA, NP, NA, MP and NS are represented in Appendix 2 Figures 20–27.

References

1. De Jong MCM, Diekmann O, Heesterbeek H. How does transmission of infection depend on population size. In: Mollison, D, editor. Epidemic models: their structure and relation to data. Cambridge: Cambridge University Press; 1995. p. 84–94.

2. European Food Safety Authority, European Centre for Disease Prevention and Control, European Reference Laboratory for Avian Influenza, Adlhoch C, Fusaro A, Gonzales JL, Kuiken T, Marangon S, Niqueux É, Staubach C, Terregino C, Aznar I, Muñoz Guajardo I and Baldinelli F, 2022. Avian influenza overview March–June 2022. EFSA J. 2022;20:e07415. https://doi.org/10.2903/j.efsa.2022.7415

3. Troost G, Boele A. Trektellen.org–store, share and compare migration data. Bird Census News. 2019;2019:17–26.

4. Lean FZX, Vitores AG, Reid SM, Banyard AC, Brown IH, Núñez A, et al. Gross pathology of high pathogenicity avian influenza virus H5N1 2021-2022 epizootic in naturally infected birds in the United Kingdom. One Health. 2022;14:100392. PubMed https://doi.org/10.1016/j.onehlt.2022.100392

Appendix 2 Table 1. Overview of dead Sandwich terns tested for avian influenza by PCR with outcome and virus sequence number if sequencing was performed

Date found	Location found	Type	X	Y	Number tested for AI by PCR	Number H5N1 positive in PCR	Virus sequence number
30 May ‘22	Colony 4	Colony outskirts bird	4.763	53.996	5	5	ST01
31 May ‘22	Colony 6*	Colony bird	4.154	51.799	7	6†	ST02
3 June ‘22	Colony 6	Colony bird	4.154	51.800	3	3	ST03-ST04
4 June ‘22	Den Oever	Away from colony	5.030	52.941	1	1	ST05
5 June ‘22	Maasvlakte	Away from colony	3.981	51.927	1	1	
5 June ‘22	Strand Ijmuiden	Away from colony	4.556	52.452	1	1	
6 June ‘22	Hellevoetsluis	Away from colony	4.119	51.826	1	1	ST06
6 June ‘22	Zandvoort	Away from colony	4.527	52.369	1	1	ST07
6 June ‘22	Stellendam	Away from colony	4.060	51.813	4	4	ST08-ST11
6 June ‘22	Colony 4	Colony outskirts bird	4.786	53.004	1	1	
7 June ‘22	Colony 4	Colony outskirts bird	4.755	53.011	1	1	
8 June ‘22	Egelsmanplaat‡	Away from colony	6.059	53.472	1	1	ST12
8 June ‘22	Renesse	Away from colony	3.722	51.739	3	3	
9 June ‘22	Den Haag	Away from colony	4.214	52.067	1	1	ST13
11 June ‘22	Colony 3§	Colony bird	4.898	53.086	3	3	ST14
12 June ‘22	Den Oever	Away from colony	5.030	52.941	2	2	ST15-ST16
13 June ‘22	Ouddorp	Away from colony	3.860	51.813	3	3	ST17
13 June ‘22	Colony 3	Colony bird	4.897	53.087	2	2	ST18
15 June ‘22	Den Andel	Away from colony	6.525	53.424	1	1	
16 June ‘22	Colony 9	Colony bird	3.518	51.400	2	2	ST19-ST20
Totals					44	43	

*Necropsy with histopathology and immunohistochemistry performed on 2 chicks, 1 PCR-positive and another PCR-negative. AI, avian influenza.
†The negative case was one of the necropsied chicks.
‡Necropsy with histopathology and immunohistochemistry on 1 adult bird.
§Necropsy with histopathology and immunohistochemistry performed on 3 adult birds.

Appendix 2 Table 2. Identification information and the results of the virological analysis for the 4 adult and 2 chick Sandwich terns submitted for necropsy

Id	Age group	Location found	Date found dead	Date necropsied	M-PCR trachea (Ct*)	M-PCR cloaca (Ct)	HPAI subtype	Sequence number
A1	Adult	Engelsmanplaat	8 June ‘22	14 June ‘22	30.27	23.15	H5N1	ST12
A2	Adult	Colony 3 (Wagejot)	11 June ‘22	14 June ‘22	20.99	18.72	H5N1	ST14
A3	Adult	Colony 3 (Wagejot)	11 June ‘22	14 June ‘22	23.66	22.99	H5N1	
A4	Adult	Colony 3 (Wagejot)	11 June ‘22	14 June ‘22	23.05	20.52	H5N1	
C1	Chick (large)	Colony 6 (Slijkplaat)	31 May ‘22	1 June ‘22	35.75	25.75	H5N1	
C2	Chick (small)	Colony 6 (Slijkplaat)	31 May ‘22	1 June ‘22	40.20	Nd†	Nd	

*Ct, cycle threshold.
†Nd, not detectable.

Appendix 2 Table 3. Gross pathology and histopathology results for the 4 adult and 2 chick Sandwich terns submitted for necropsy*

Id	Sex	Condition	Stomach contents	Other	Histopathologic summary
A1	M	Normal	Empty	No changes in the pancreas or the brain. Spleen not enlarged.	Mild hyperemia in cerebrum and cerebellum, no infiltrates. Lung edema, pneumoconiosis, possibly edema in the parabronchial lumen and air trapped in capillary lumen. Spleen np†. Other organs severe autolysis.
A2	F	Normal	Empty	No changes in the pancreas or the brain. Spleen not enlarged.	Mild hyperemia in cerebrum and cerebellum, no infiltrates. Hyperemic lungs, edema in the parabronchial lumen, pneumoconiosis. Focal extensive acute necrosis in the pancreas. Spleen np. Uric acid congestion in tubular lumina of kidney with minimal mineralization. Ovarium no lesions.
A3	F	Normal	Empty	No changes in the pancreas or the brain. Spleen not enlarged.	Possibly infiltrates in glandular tissue next to eye. Mild hyperemia in cerebrum and cerebellum, no infiltrates. Hyperemic lungs, edema in the parabronchial lumen, pneumoconiosis. Non suppurative enteritis duodenum. Extensive acute necrosis of the exocrine pancreas. Liver multifocal necrosis. Spleen np. Ovarium no lesions.
A4	M	Normal	Empty	No changes in the pancreas or the brain. Spleen not enlarged.	Necrosis, hemorrhage, and round nucleated cells with fibrin in glandular nasal/sinus tissue. Mild hyperemia in cerebrum and cerebellum, no infiltrates. Lung edema in the parabronchial lumen, pneumoconiosis, non-suppurative infiltrates interstitially. Extensive acute necrosis of the exocrine pancreas. Few lymphocytes in the lamina propria in the duodenum. Spleen autolysis with mixed population of lymphocytic and heterophilic granulocytes, few macrophages with hemosiderin. Adult trematodes in the collecting tubes of the kidney.
C1	Nd	Nd	Empty	Juvenile bird	Subepthelial lymphoid infiltrates in sinus, acute fibrinous pneumonia, non-suppurative gastritis, other organs severe autolysis.
C2	Nd	Nd	Empty	Juvenile bird	Necrotic heterophilic epidermitis next to beak, subepethelial lymphoid and heterophilic infiltrates in sinus, hepatic vacuolization, spleen np, other organs no significant changes.

*Nd, not detectable; Np, not present.

Appendix 2 Table 4. Immunohistochemistry results for the 4 adult and 2 chick Sandwich terns submitted for necropsy*

Id	Duodenum	Pancreas	Nasal/Sinus tissue	Lung	Brain	Other
A1	1	1	Np	0	0	0
A2	1	0	0	0	0	0
A3	1*	1*	0	0	0	0
A4	1	1	1	1	0	0
C1	0	0	0	0	0	0
C2	0	0	0	0	0	0

*Included in Appendix 2 Figure 18. Np, not present.

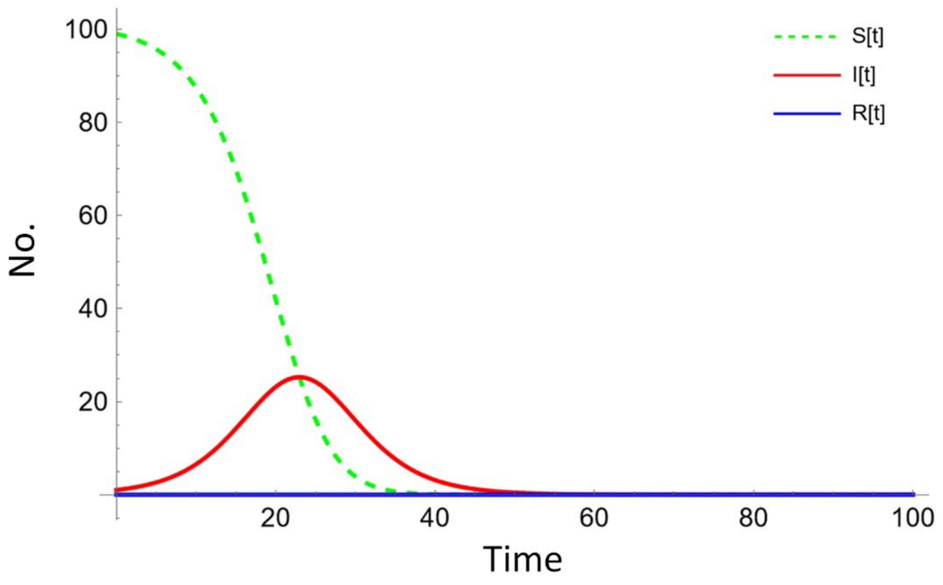

Appendix 2 Figure 1. The dynamics of the infection in a local population with infection-fatality rate = 0.0. S are susceptible (naive) birds, I are infected birds, and R are recovered (immune) birds.

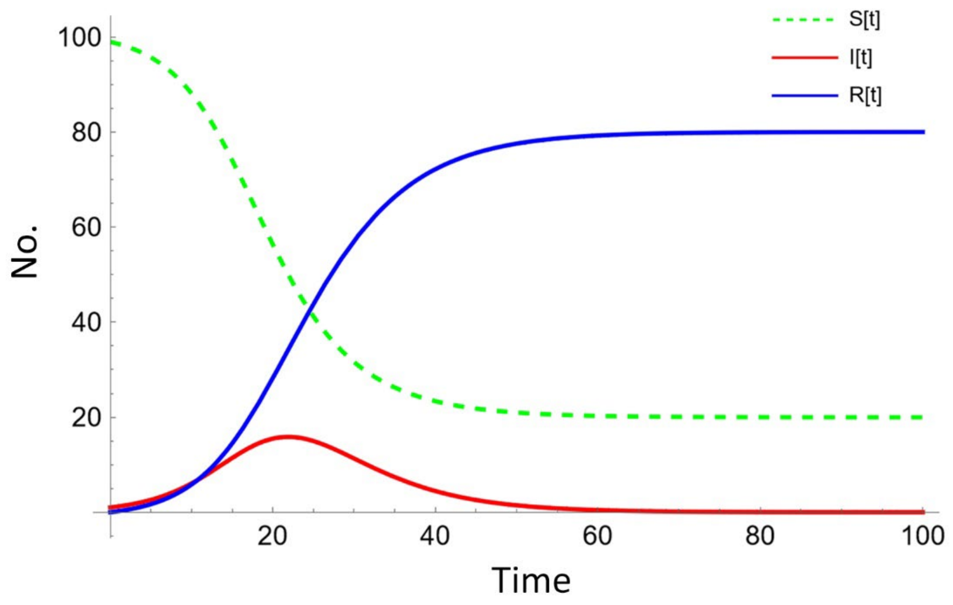

Appendix 2 Figure 2. The dynamics of the infection in a local population with infection-fatality rate = 1.0. S are susceptible (naïve) birds, I are infected birds, and R are recovered (immune) birds.

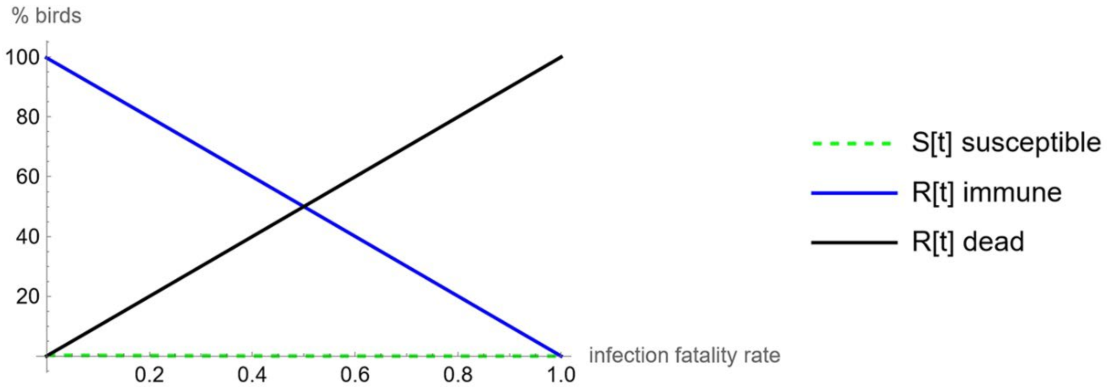

Appendix 2 Figure 3. The final size for R_0 = 6.0

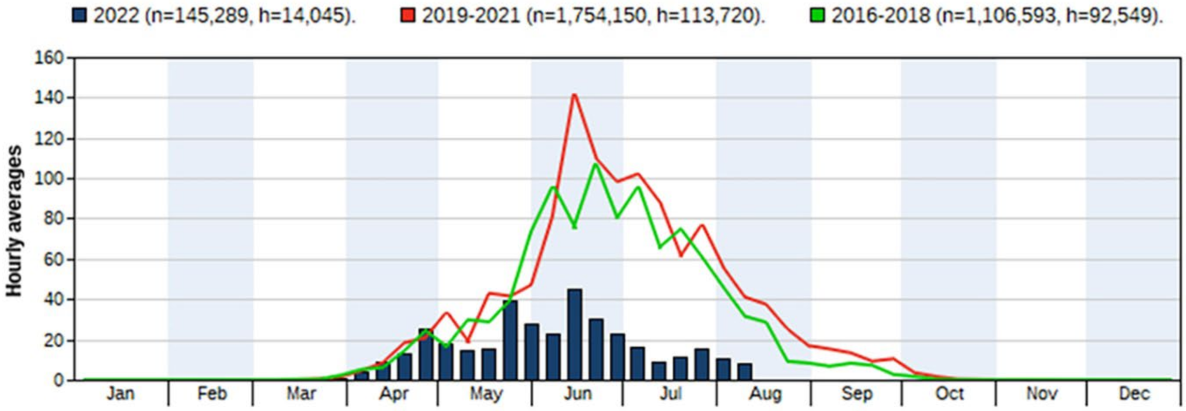

Appendix 2 Figure 4. Alongshore movements of Sandwich terns at coastal observation points in the Netherlands in 2022 (until August 10, 2022) compared to 2019–2021 and 2016–2018.

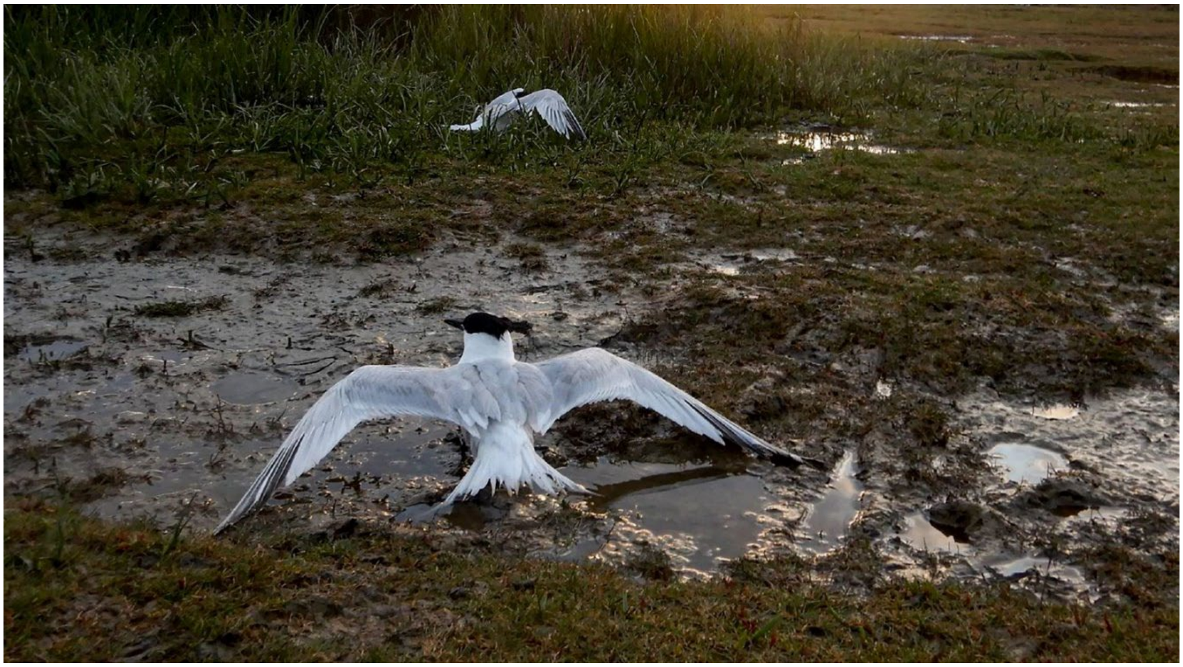

Appendix 2 Figure 5. Diseased Sandwich terns unable to fly away. HPAI H5N1 outbreak, Breeding Colony 4 (Petten) outskirts, June 12, 2022. Photo by Susanne Kühn.

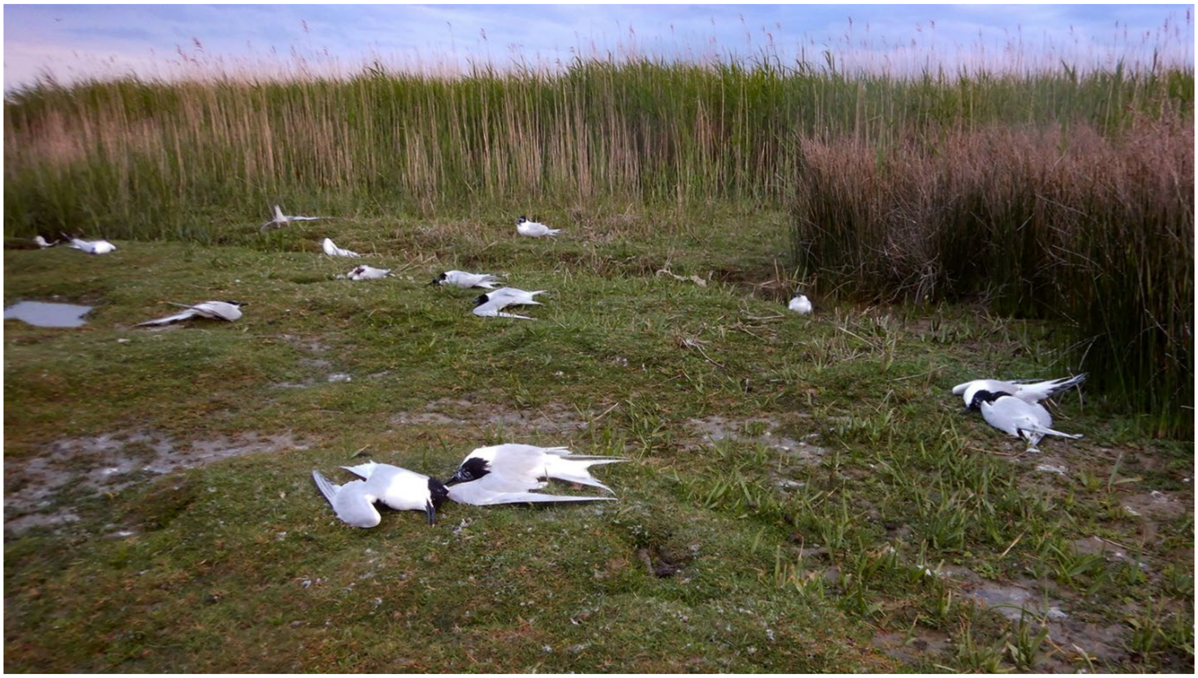

Appendix 2 Figure 6. Diseased lethargic and dead Sandwich terns. HPAI H5N1 outbreak, Breeding Colony 4 (Petten) outskirts, June 12, 2022. Photo by Susanne Kühn.

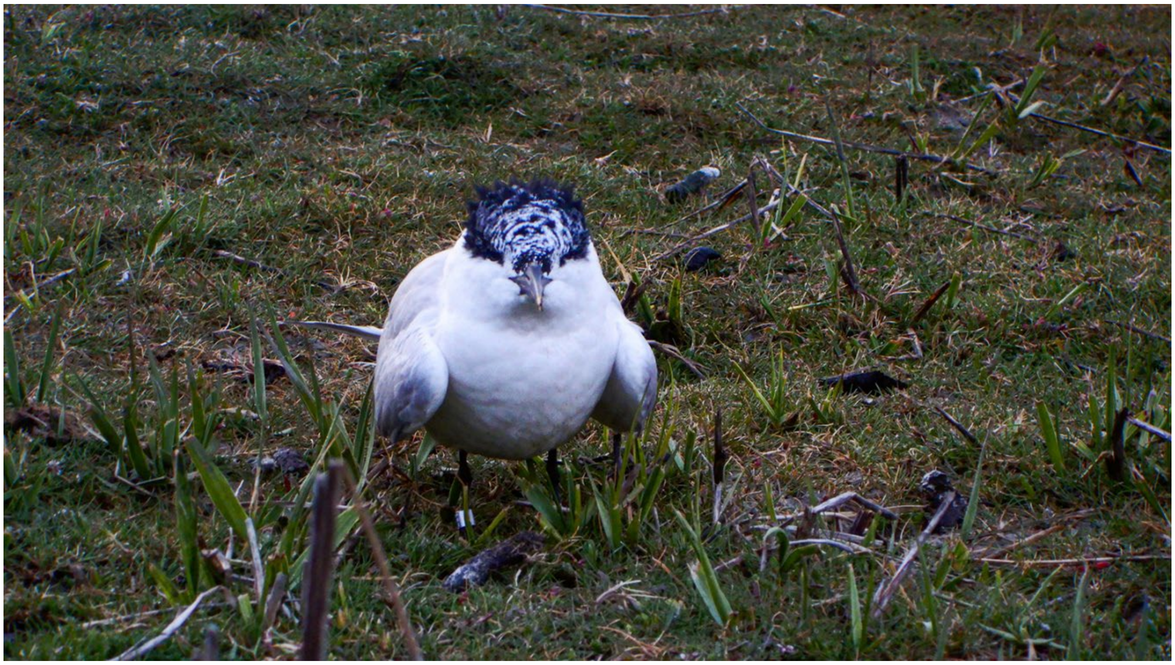

Appendix 2 Figure 7. Diseased lethargic Sandwich tern. HPAI H5N1 outbreak, Breeding Colony 4 (Petten) outskirts, June 12, 2022. Photo by Susanne Kühn.

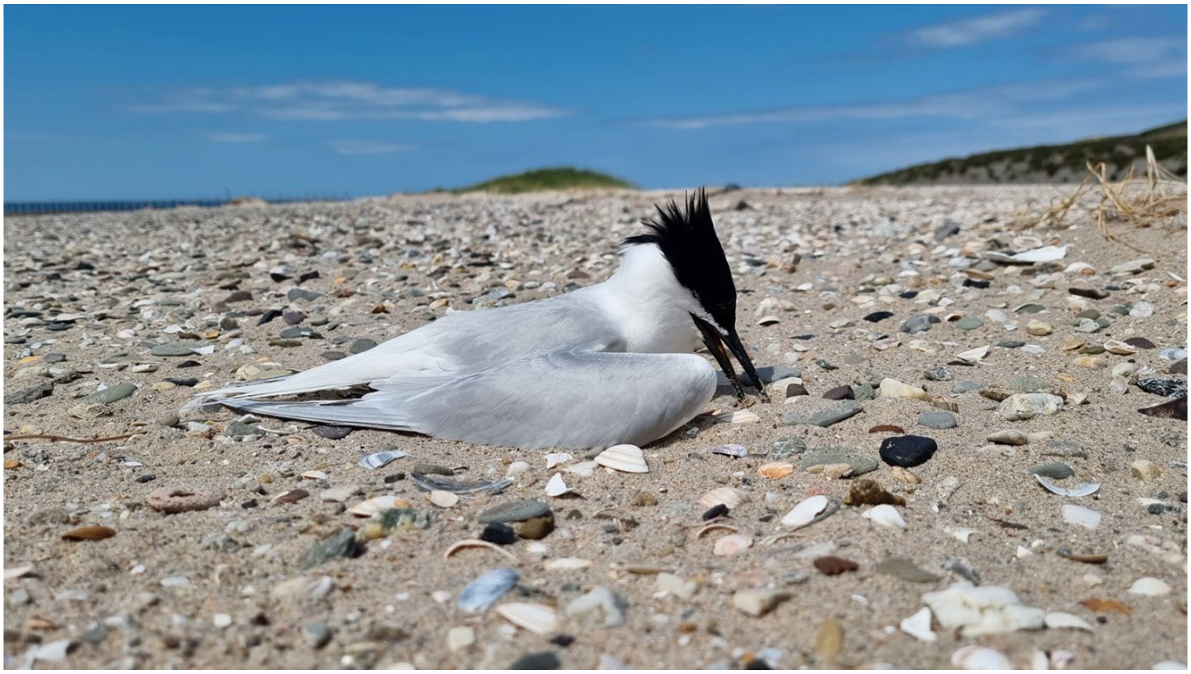

Appendix 2 Figure 8. Diseased lethargic Sandwich tern, wings hanging, respiratory distress.

HPAI H5N1 outbreak, away from colonies, June 14, 2022. Photo by Sander J. Lilipaly.

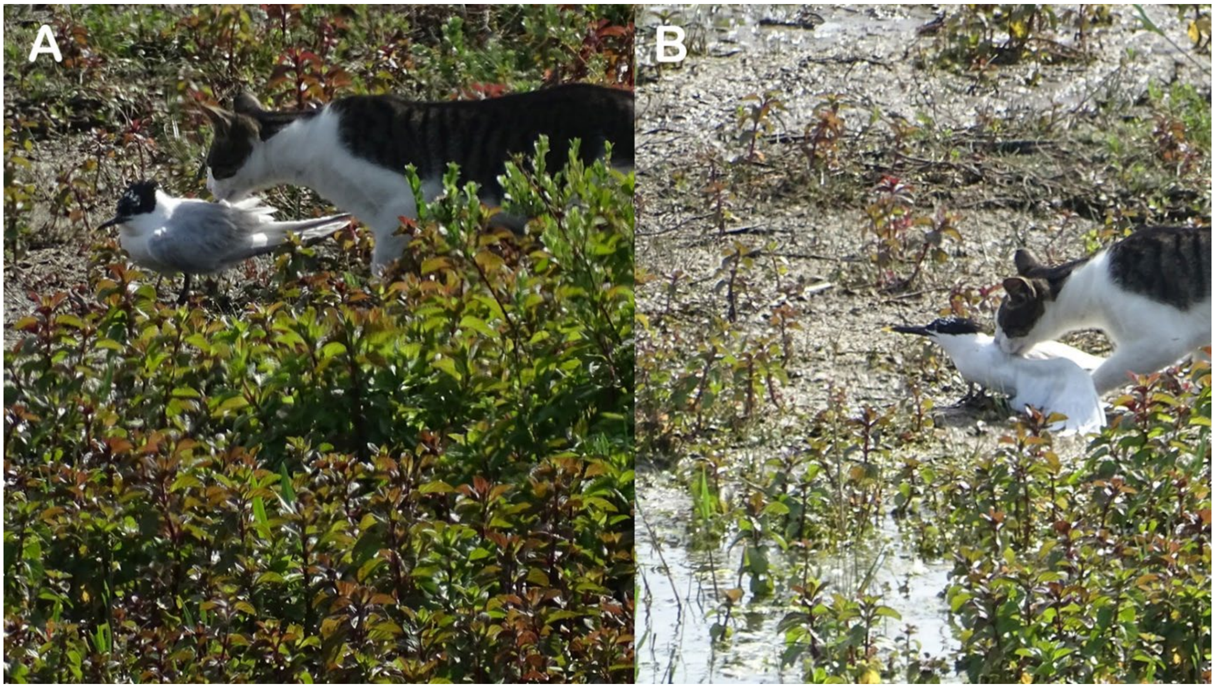

Appendix 2 Figure 9. Diseased lethargic Sandwich tern, unable to flee the cat. HPAI H5N1 outbreak, vicinity of Breeding Colony 2 (Steenplaat), June 18, 2022. Photos by Wilma Booij.

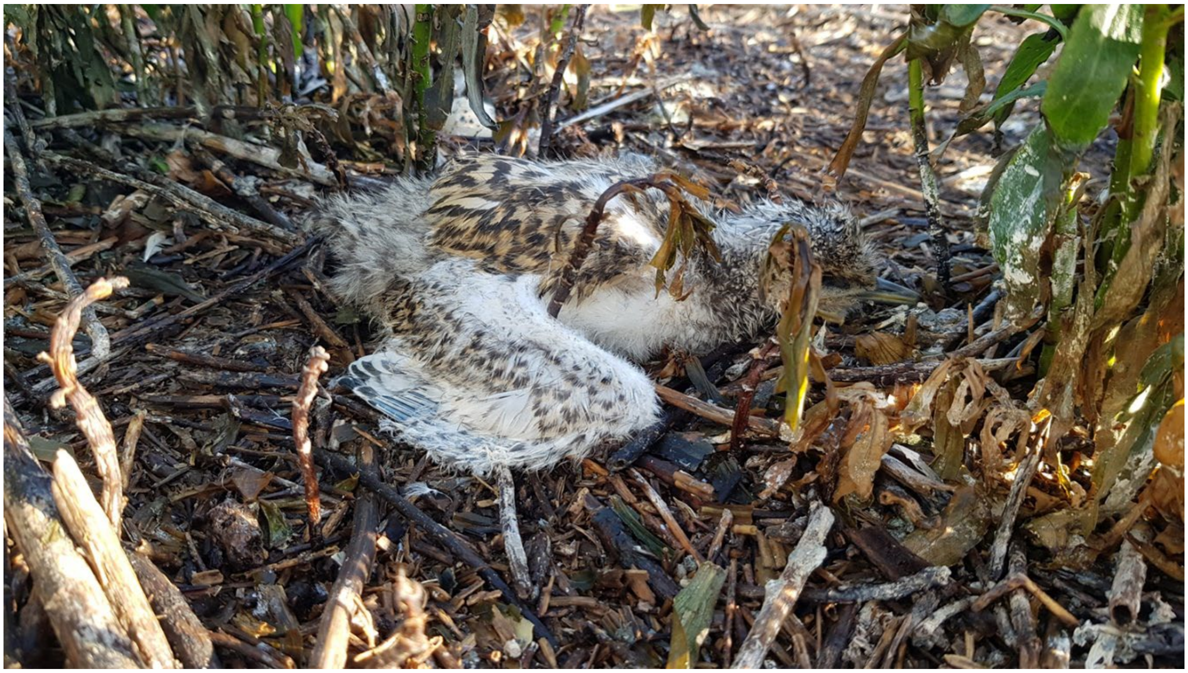

Appendix 2 Figure 10. Diseased Sandwich tern fledgling, unable to flee, wings hanging wide aside. HPAI H5N1 outbreak, Breeding Colony 6 (Slijkplaat), June 6, 2022. Photo by Mónika Z. Ballmann.

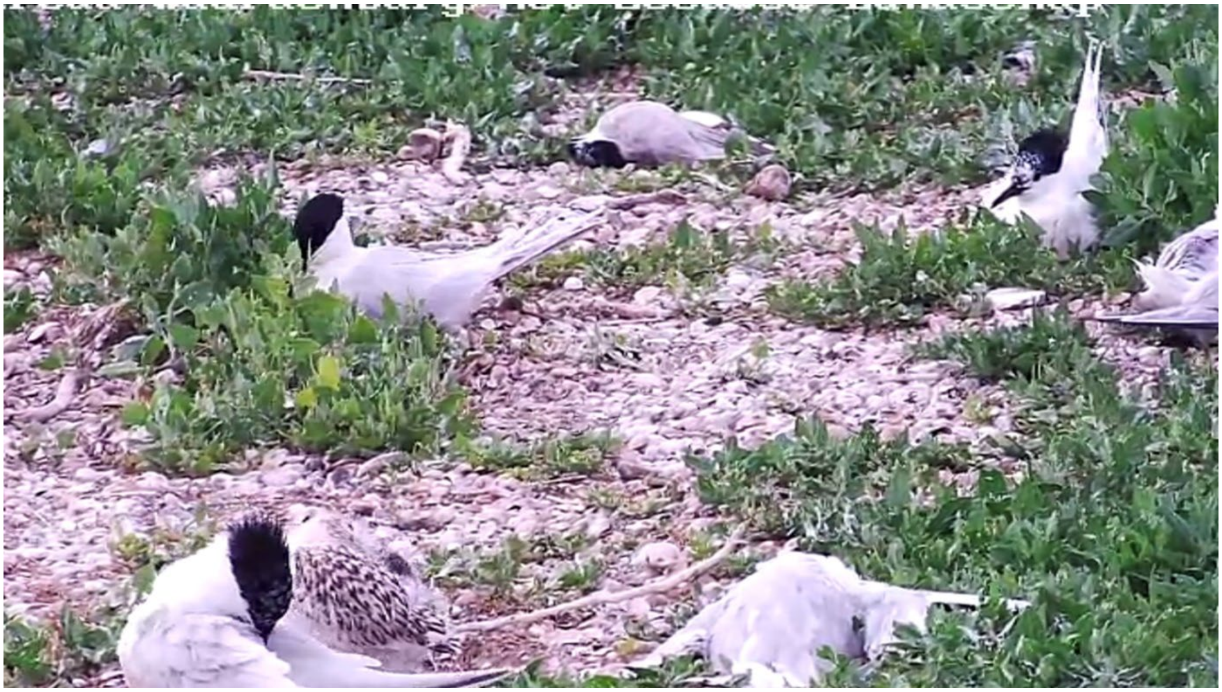

Appendix 2 Figure 11. Sandwich terns, breeding among the dead. HPAI H5N1 outbreak, Breeding colony 9 (Waterdunen), June 2022. Webcam image Bureau Waardenburg/Het Zeeuws Landschap.

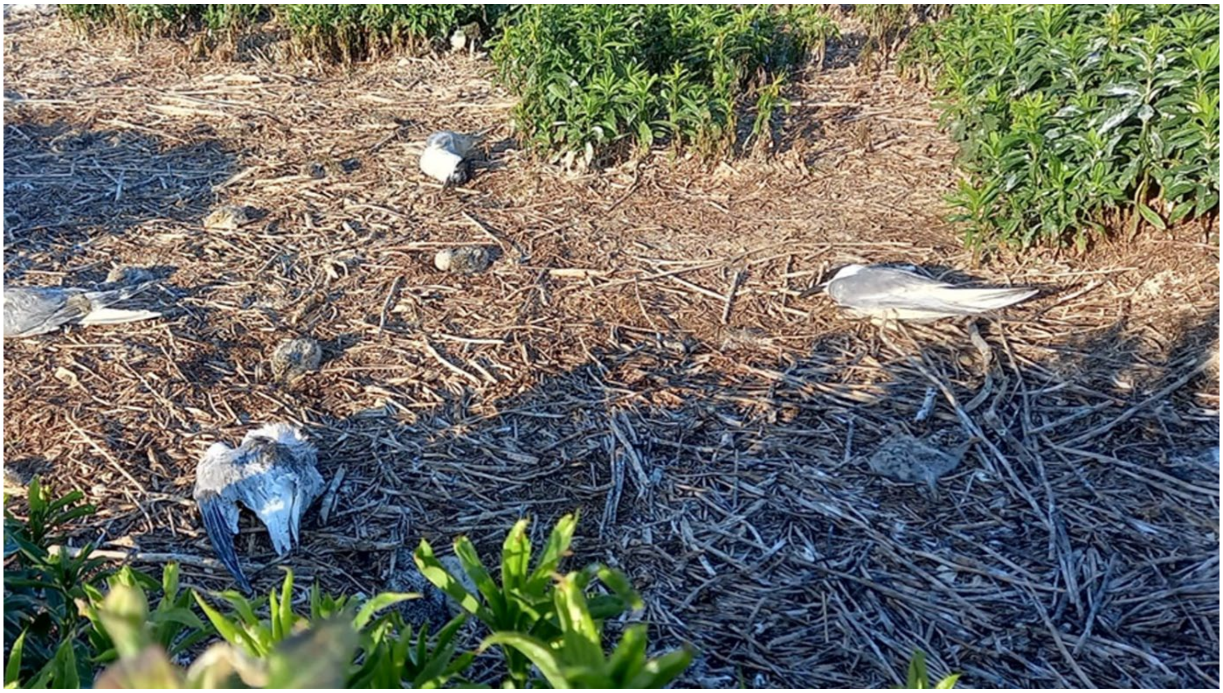

Appendix 2 Figure 12. Sandwich terns dead on nests, some live chicks (these sometimes then starved to death – see pathology). HPAI H5N1 outbreak, Breeding Colony 6 (Slijkplaat), June 4, 2022. Photo by Ronald in ’t Veld.

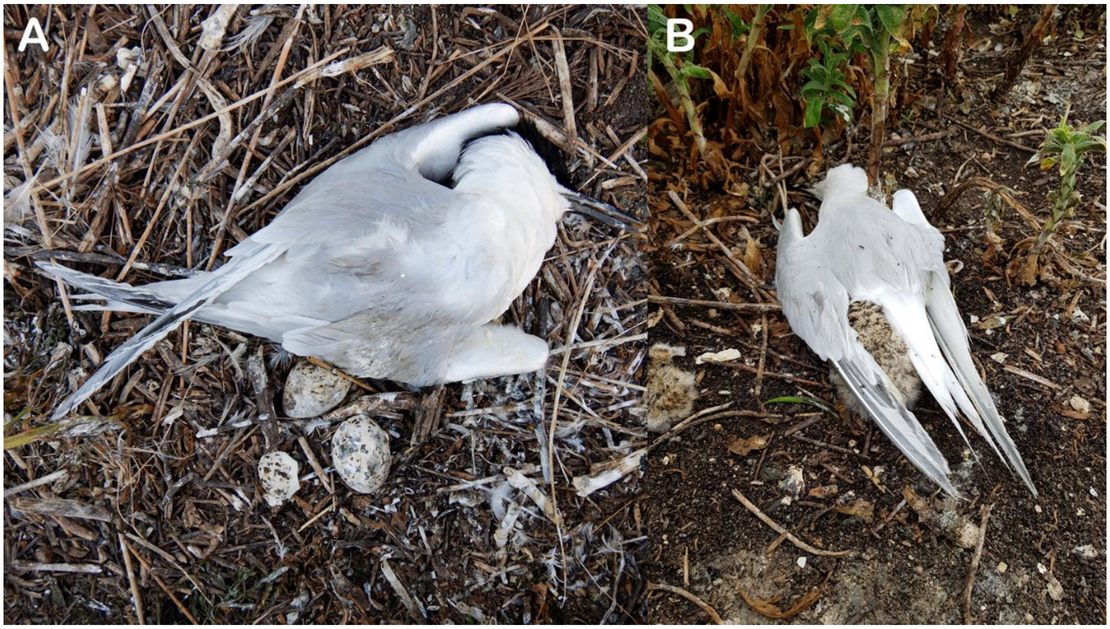

Appendix 2 Figure 13. Sandwich terns, dead on nest. HPAI H5N1 outbreak. A) Breeding Colony 3 (Wagejot), June 15, 2022. B) Breeding Colony 6 (Slijkplaat), June 6, 2022. Photo A by Susanne Kühn, photo B by Mónika Z. Ballmann.

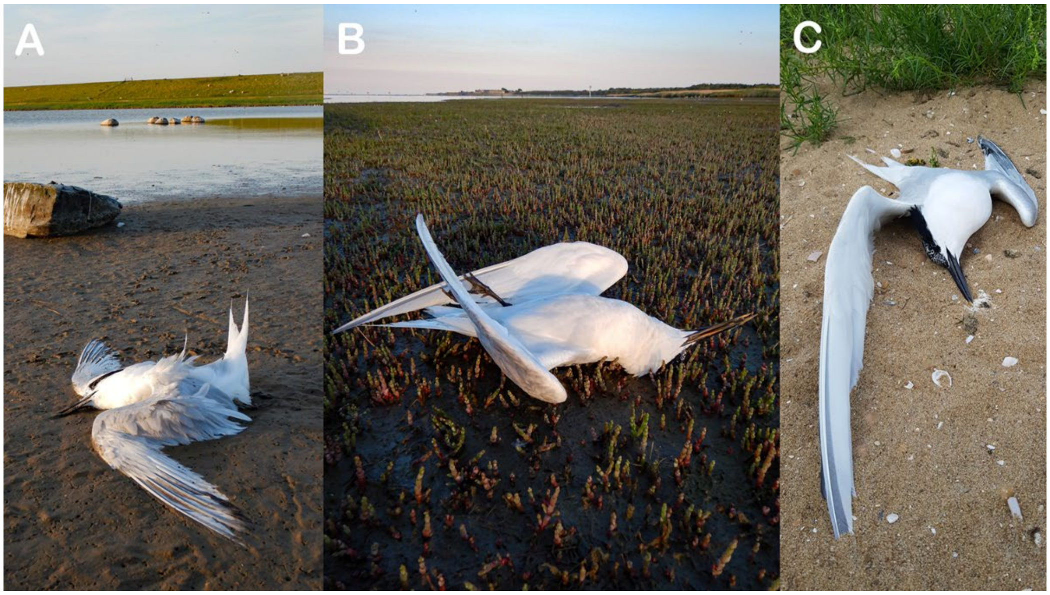

Appendix 2 Figure 14. Dead Sandwich terns with tail still in opisthotonos position (A), birds in flipped over distorted position (B, C). HPAI H5N1 outbreak, Breeding colony 4 (Petten) outskirts, June 14, 2022 (A) and June 17, 2022 (B) and Breeding Colony 6 (Slijkplaat), June 10, 2022 (C). Photos A and B by Susanne Kühn, photo C by Mónika Z. Ballmann.

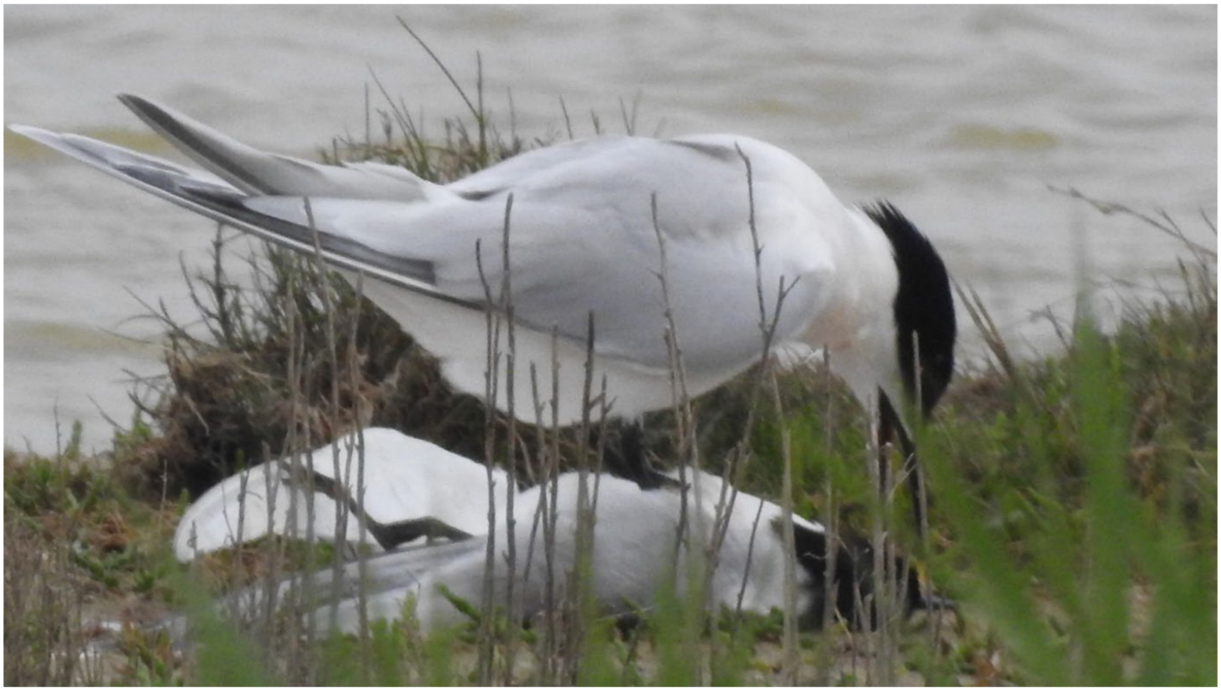

Appendix 2 Figure 15. Necrophilia may have enhanced transmission. HPAI H5N1 outbreak, Breeding Colony 3 (Wagejot), June 5, 2022. Photo by Mardik Leopold.

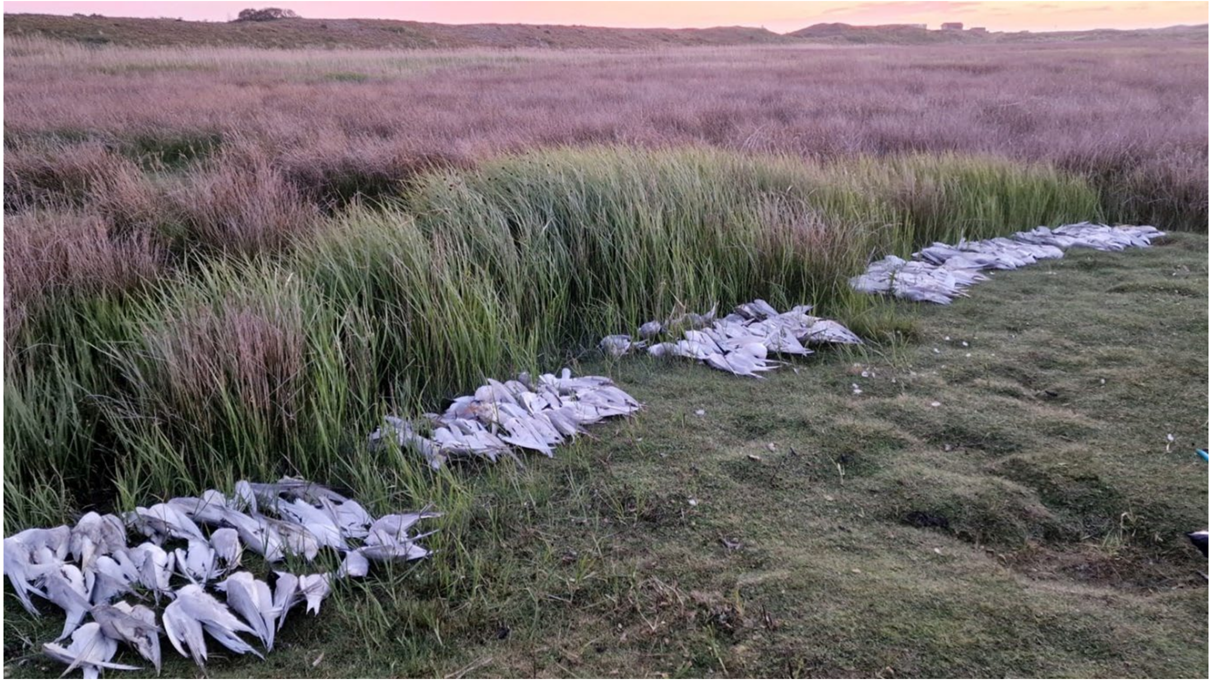

Appendix 2 Figure 16. Sandwich tern carcass removal. HPAI H5N1 outbreak, outskirts of Breeding Colony 4 (Petten), 4 June 4, 2022. Photo by Marc Plomp.

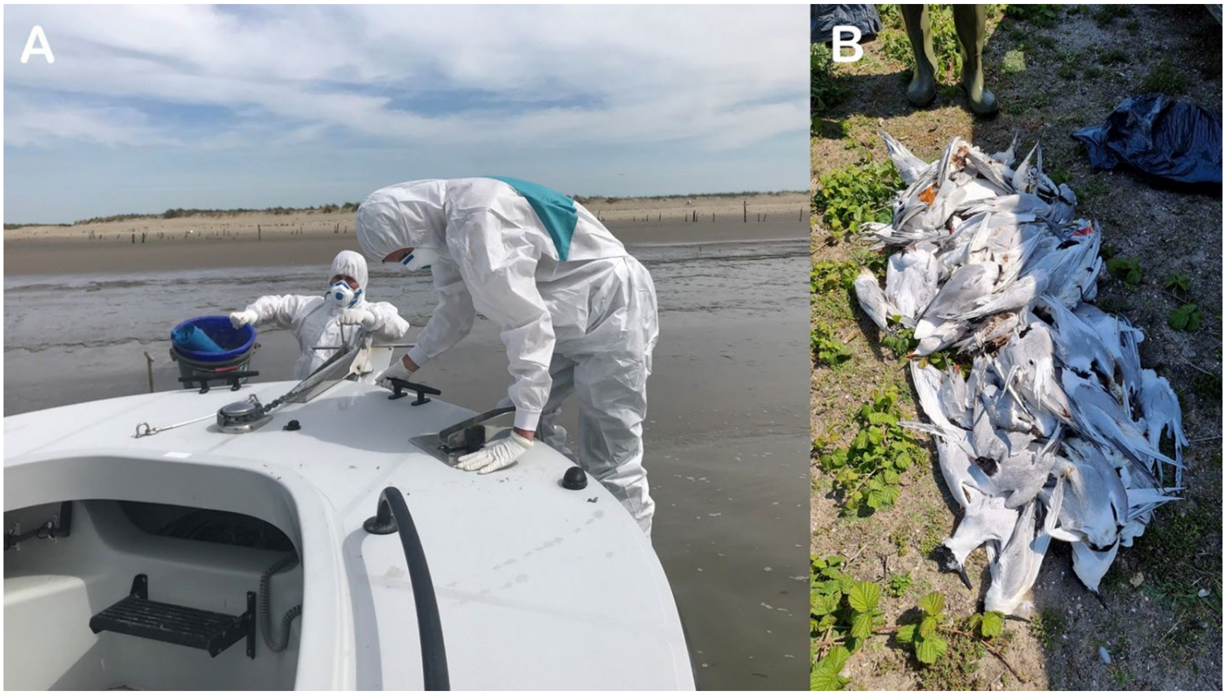

Appendix 2 Figure 17. Sandwich tern carcass removal. HPAI H5N1 outbreak, Breeding Colony 10 (Hooge Platen), June 21, 2022 (A) and away from colonies (Scheelhoek), June 15, 2022. Photo A by Fred Schenk, photo B by Mónika Z. Ballmann.

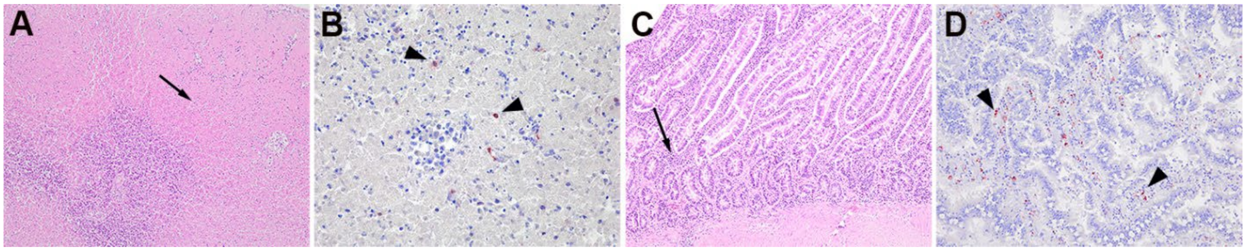

Appendix 2 Figure 18. HPAI related histopathology and viral protein expression: A) Extensive severe acute pancreas necrosis with loss of cellular detail (arrow) evaluated with hematoxylin and eosin (HE) stain, objective 20x; B) multifocal positive staining of individual epithelial cells in pancreas (arrowhead), immunohistochemistry (IHC), objective 40x; C) mild to moderate lymphoplasmacytic duodenitis (arrow) with viral antigen expression in epithelial cells (arrowhead); D), objective 20x.

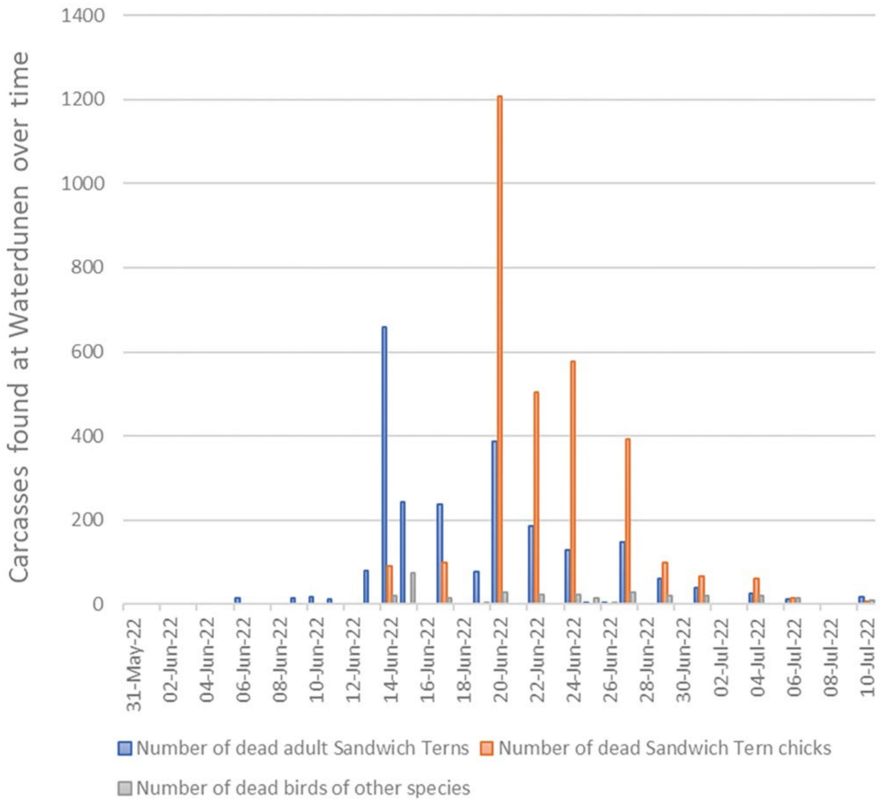

Appendix 2 Figure 19. Adult Sandwich tern mortality peaked before chick mortality in Breeding Colony 9 (Waterdunen).

PB2

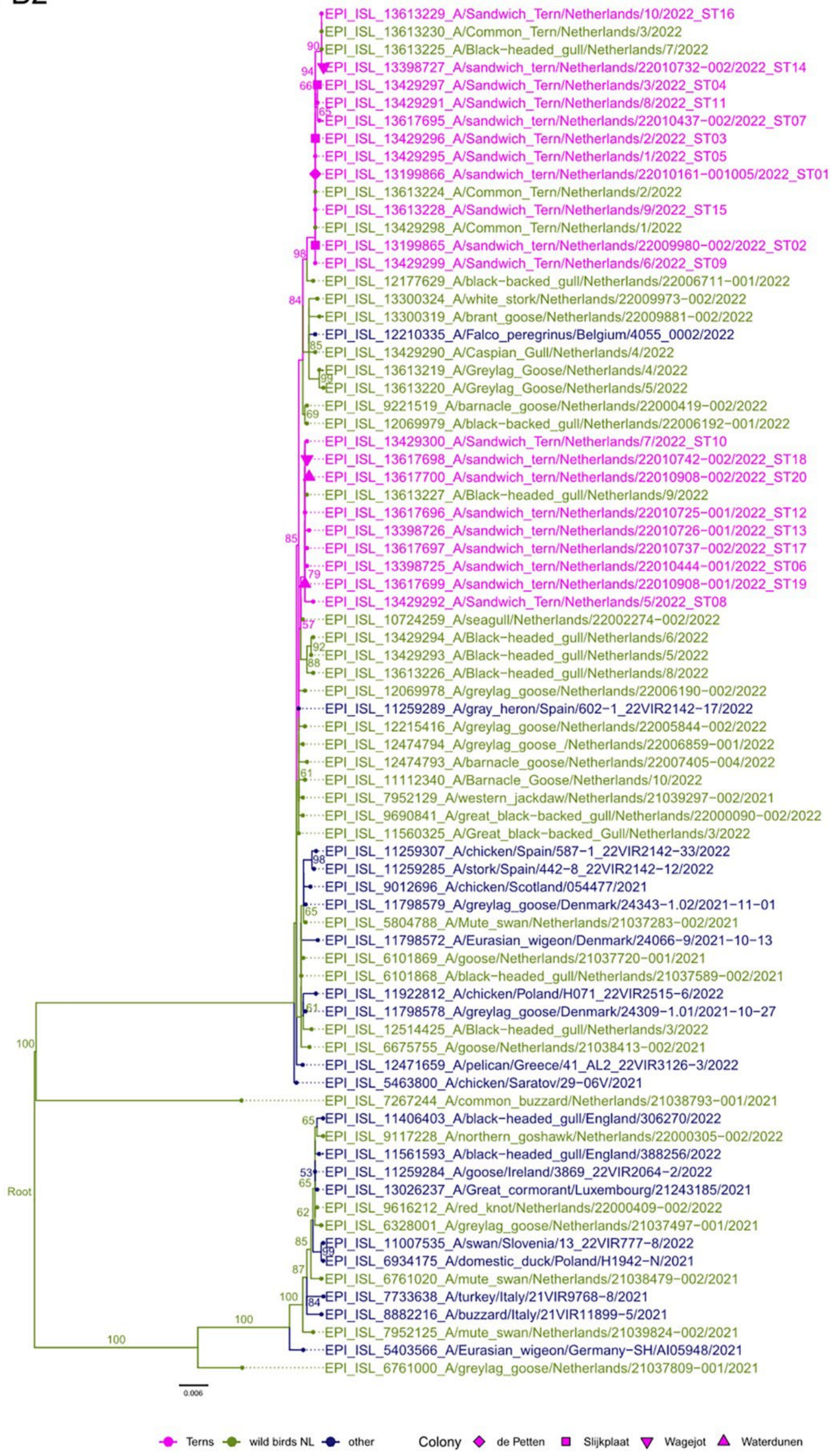

Appendix 2 Figure 20. The maximum-likelihood (ML) tree for PB2

PB1

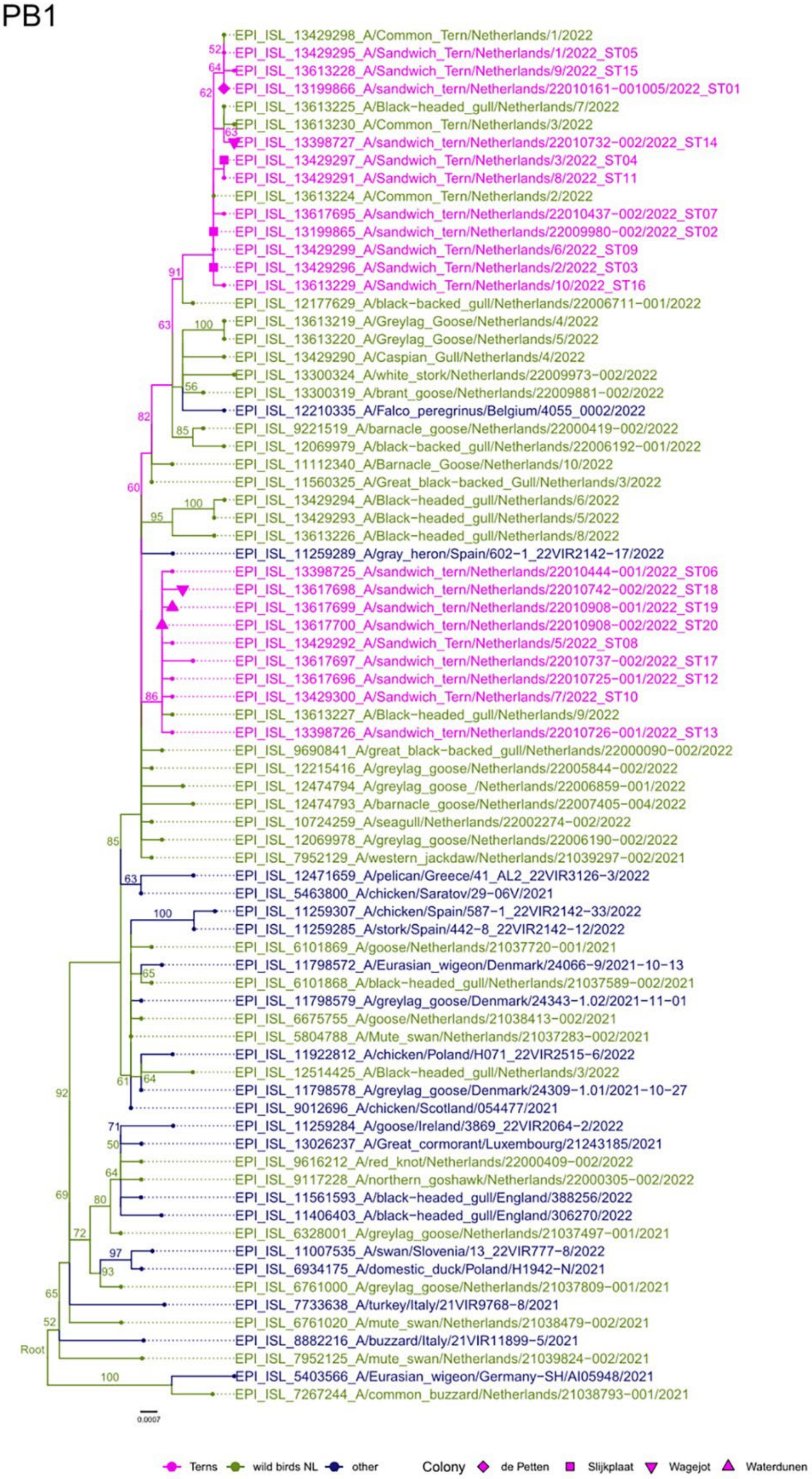

Appendix 2 Figure 21. The maximum-likelihood (ML) tree for PB1

PA

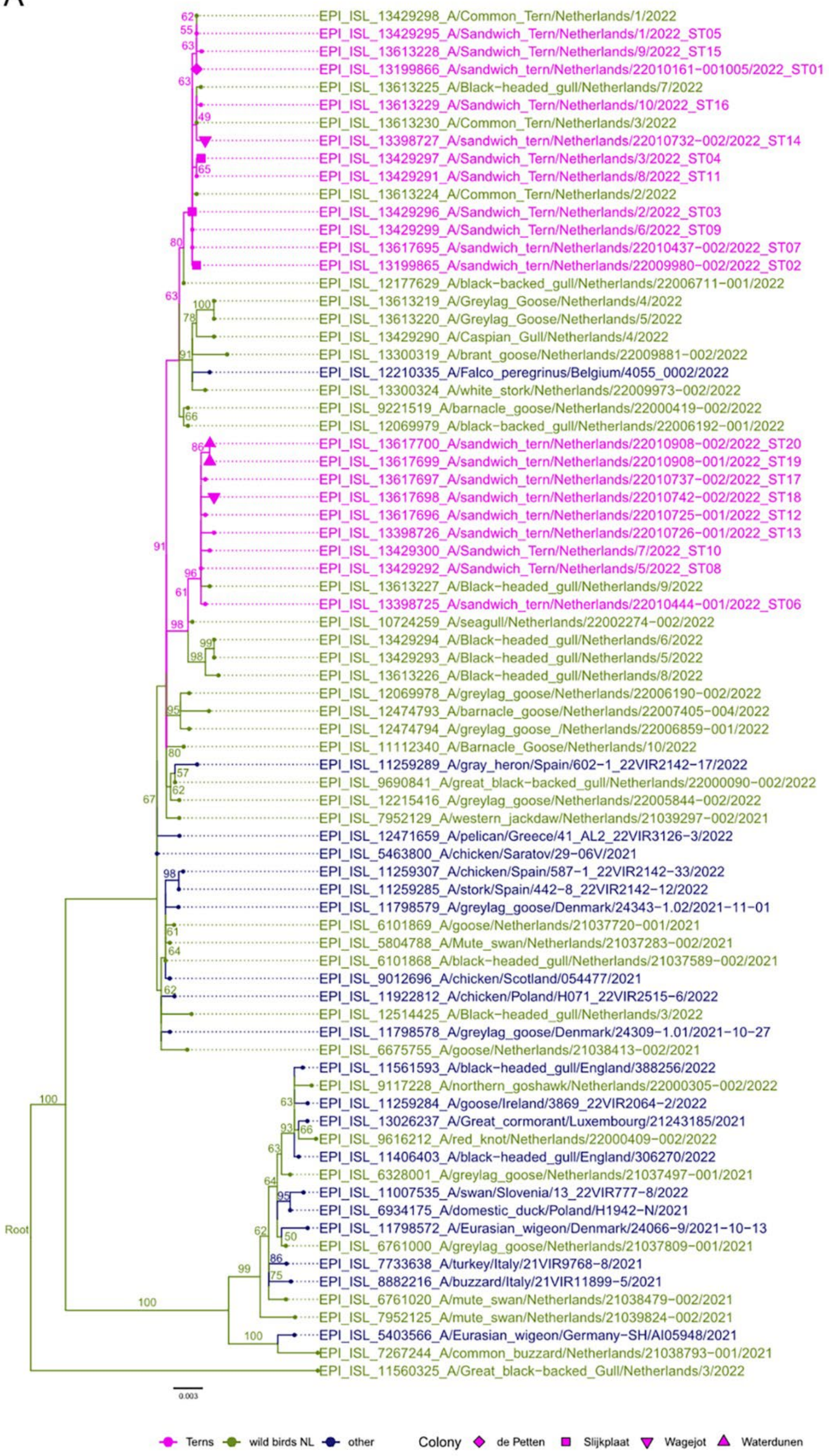

Appendix 2 Figure 22. The maximum-likelihood (ML) tree for PA

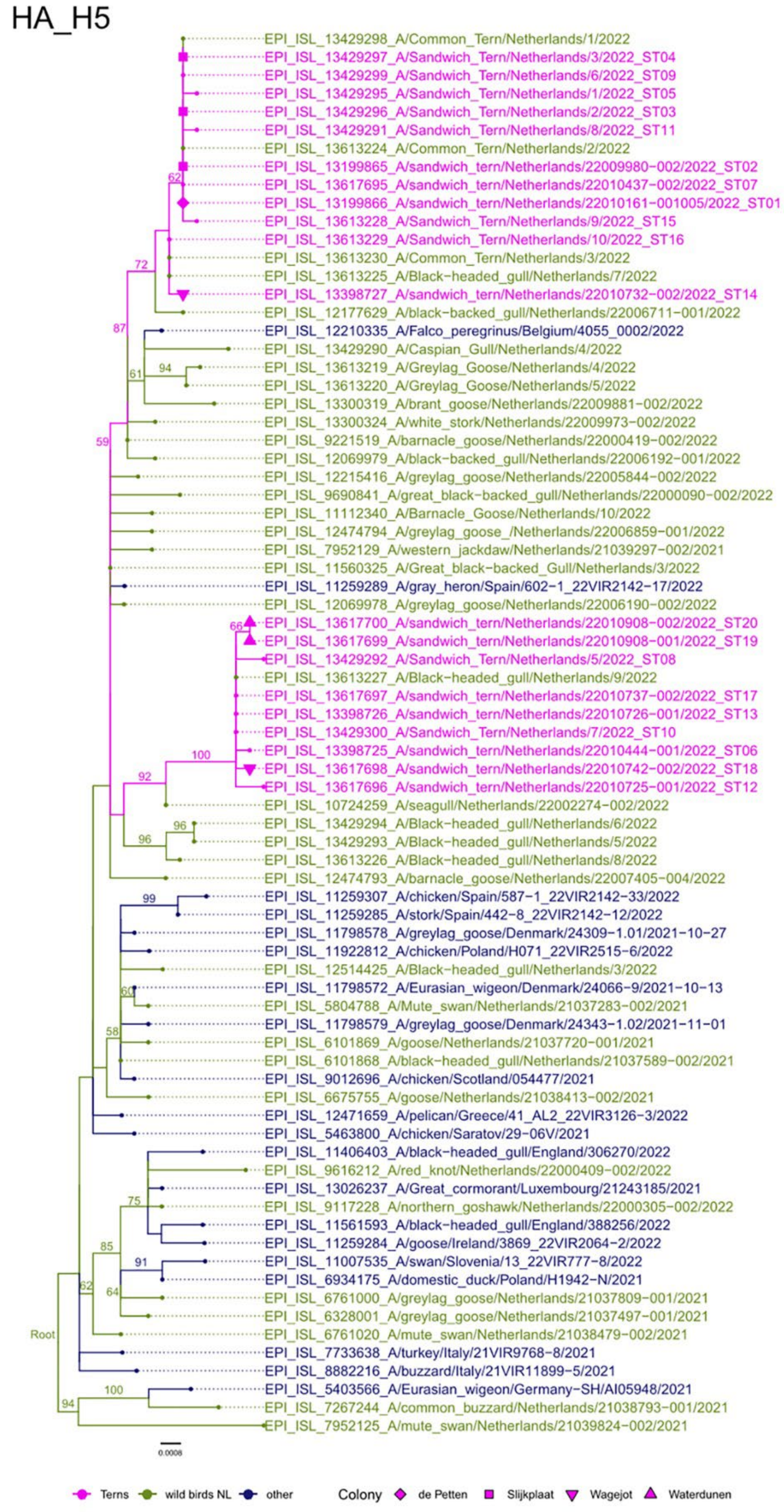

Appendix 2 Figure 23. The maximum-likelihood (ML) tree for HA

NP

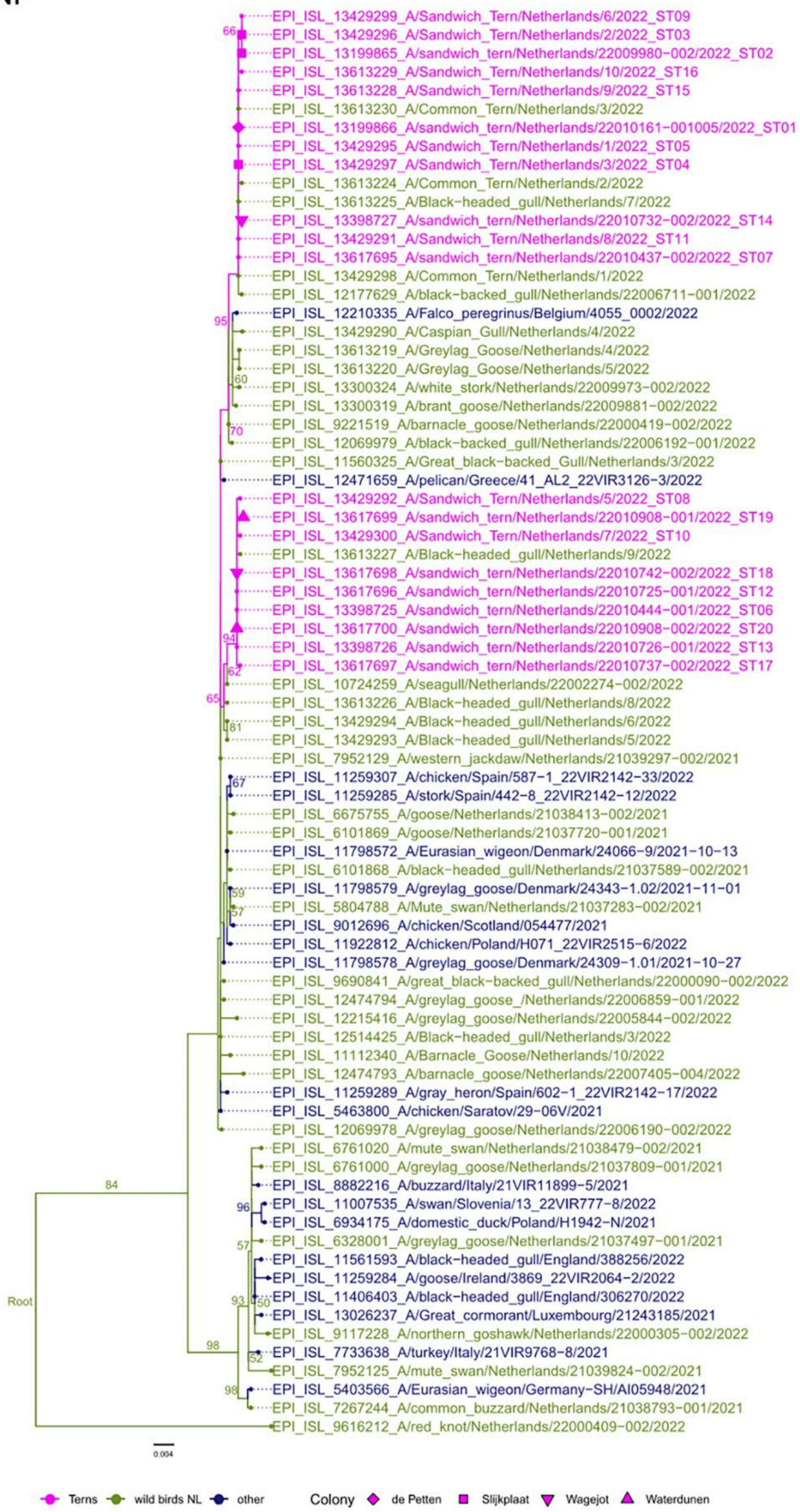

Appendix 2 Figure 24. The maximum-likelihood (ML) tree for NP

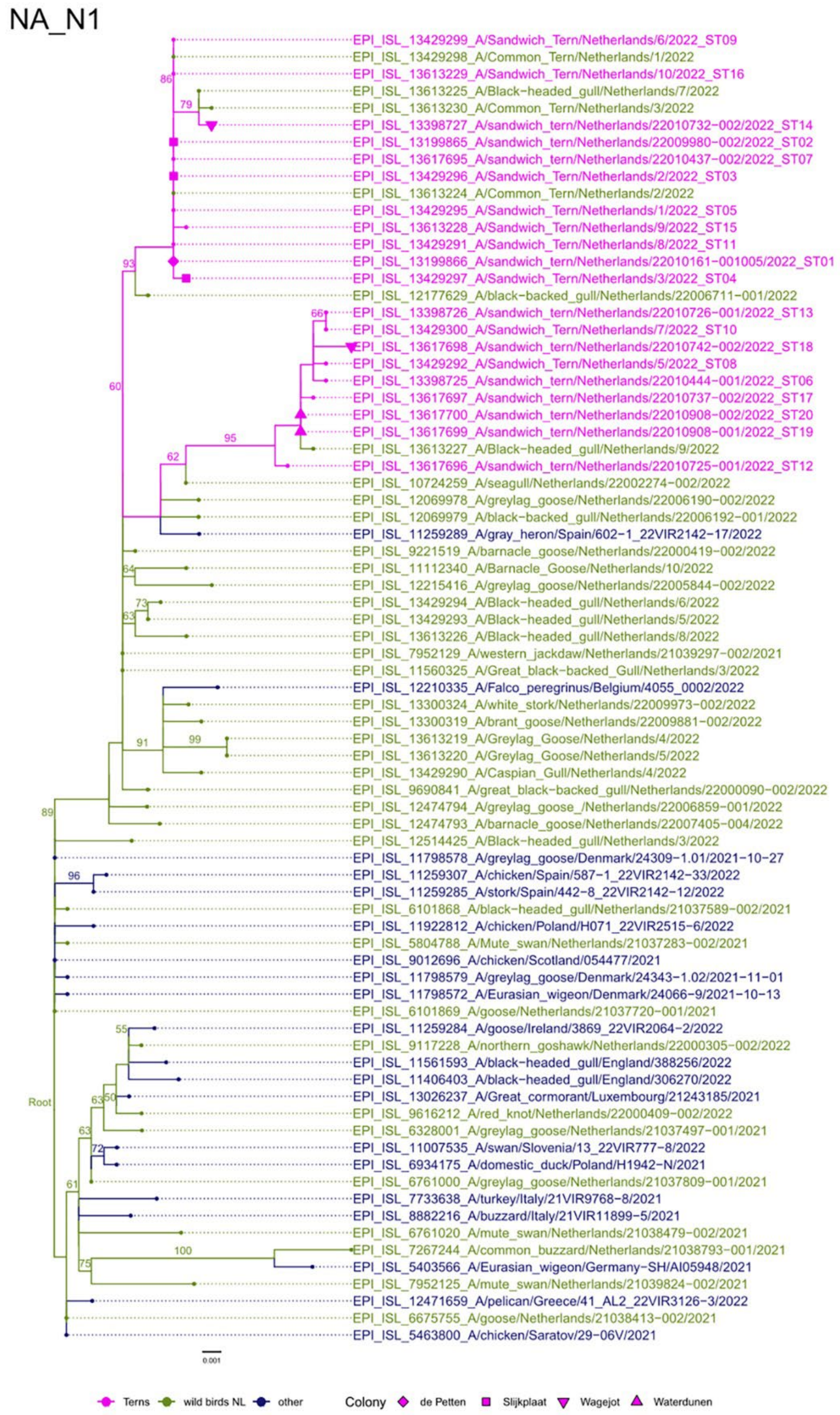

Appendix 2 Figure 25. The maximum-likelihood (ML) tree for NA

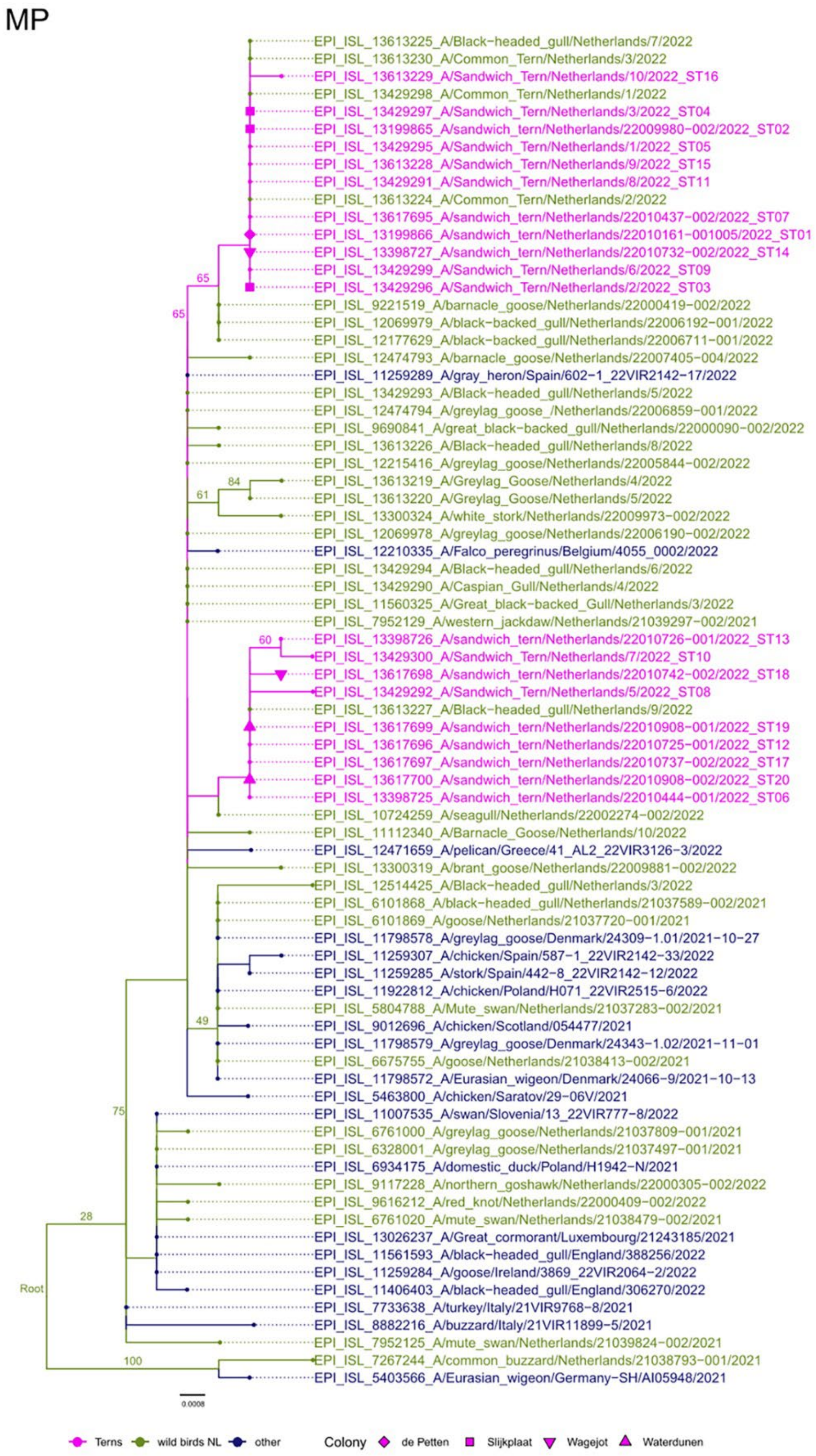

Appendix 2 Figure 26. The maximum-likelihood (ML) tree for MP

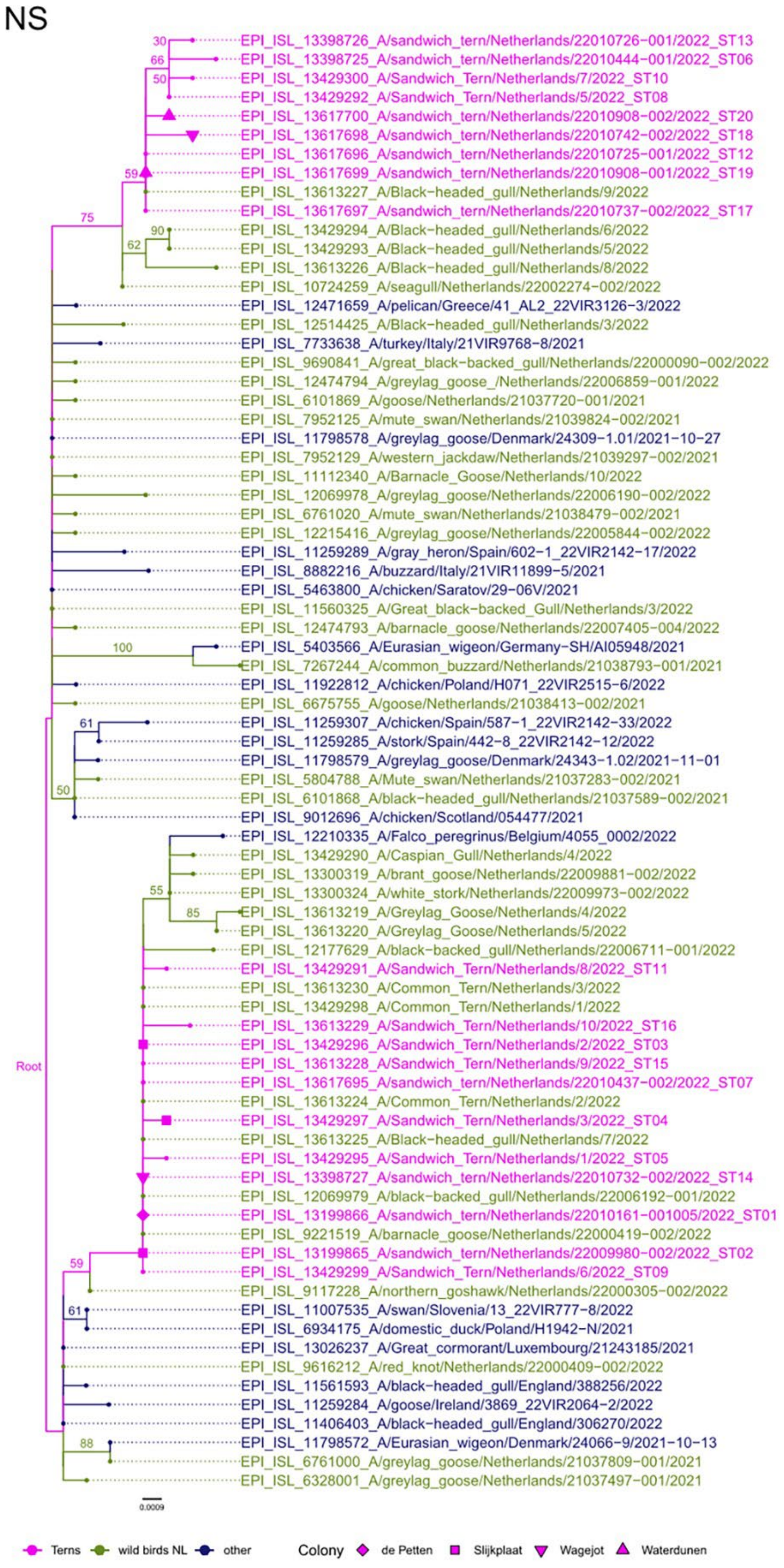

Appendix 2 Figure 27. The maximum-likelihood (ML) tree for NS
